# GPSai: A Clinically Validated AI Tool for Tissue of Origin Prediction during Routine Tumor Profiling

**DOI:** 10.1158/2767-9764.CRC-25-0171

**Published:** 2025-09-01

**Authors:** Hassan Ghani, Anthony Helmstetter, Jennifer R. Ribeiro, Todd Maney, Stephanie Rock, Rebecca A. Feldman, Jeff Swensen, Farah Abdulla, David B. Spetzler, Elena Florento, Ari M. Vanderwalde, Patricia Pittman, Milan Radovich, Jaclyn Hechtman, Casey Bales, George W. Sledge, Myra M. George, David Bryant, Jim P. Abraham, Matthew J. Oberley

**Affiliations:** Caris Life Sciences, Irving, Texas.

## Abstract

A subset of cancers present with unclear or potentially incorrect primary histopathologic diagnoses, including cancers of unknown primary (CUP). We aimed to develop and validate an artificial intelligence (AI) tool, Genomic Probability Score AI (GPSai™), which predicts tumor tissue of origin in CUP and flags potential misdiagnoses for additional workup during routine molecular testing. The GPSai model was trained on whole exome and whole transcriptome data from 201,612 cases submitted for tumor profiling at Caris Life Sciences. Retrospective (*N* = 21,549) and prospective (*N* = 76,271) validations were performed. The clinical impact was evaluated over 8 months of live testing and through physician surveys. GPSai demonstrated 95.0% accuracy in non-CUP cases and reported on tumor tissue of origin in 84.0% of CUP and 96.3% of non-CUP cases. During the initial 8 months of implementation, GPSai changed the diagnosis on 704 patients (0.88% of all profiled cases), which were supported by orthogonal evidence including imaging, IHC, mutational signatures, hallmark fusions, or viral reads. Diagnosis changes prompted changes in targeted therapy eligibility based on level 1 clinical evidence in 86.1% of cases (*n* = 606/704). A majority (89.7%; *n* = 87/97) of physician responses indicated acceptance of the GPSai results, and 53.6% (*n* = 52/97) of responses stated that the results prompted a change in treatment plan. GPSai accurately identifies tumor tissue of origin and has the potential for clinical impact in a small but meaningful subset of patients with CUP or pathologically ambiguous tumors. Our results support the integration of this AI tool into routine molecular testing to improve diagnostic accuracy and guide subsequent therapeutic decisions.

**Significance:**

Our findings show that GPSai, a deep learning–based tool, can support the identification of primary tumor sites with high accuracy in conjunction with orthogonal evidence. Its integration into routine tumor profiling furthermore allows simultaneous biomarker identification. Analysis of real-world implementation of GPSai shows that it enhances diagnostic accuracy, including resolution of CUP cases, and prompts clinically relevant therapeutic recommendation changes without requiring additional specimen.

## Introduction

Whereas a majority of metastatic tumors are readily classified based on standard pathologic and clinical evaluation, a subset is more challenging to diagnose and may present with unclear or potentially incorrect primary organ and histologic diagnoses. Among these are “cancers of unknown primary” (CUP), which compose ∼2% of cancer cases (1–4) and are associated with lack of effective treatment options and poor outcomes (1, 5–8). Metastatic tumors may also be assigned a diagnostic label but present with diagnostic discrepancies upon further evaluation. Whereas studies suggest that the rate of pathology diagnostic discrepancies is relatively low, inaccuracies in diagnosis, including tumor lineage changes, can affect treatment planning (9–17). As such, accurate diagnosis of pathologically ambiguous cancers is critical to providing effective patient care.

In recent years, artificial intelligence (AI) tools have been developed to help deal with diagnostic ambiguities and improve diagnostic accuracy. Gene expression profiling–based models for identification of tumor tissue of origin report diagnostic accuracies in the range of 73% to 96%, with varying ability to classify CUP cases (18–25). More recently, next-generation sequencing (NGS)-based genomic profiling has been used to identify tumor tissue of origin and reveal actionable targets simultaneously (26–36). The American Society of Clinical Oncology has stated the potential value of such an approach (37). However, factors limiting the clinical impact of tissue of origin classifiers include low numbers of samples or cancer types used in the training set, excessive cost of the technique, low-quality RNA for gene expression–based assays, contamination of the tumor biopsy with surrounding tissue (38), and limited tissue availability for testing (39, 40).

More broadly, there are multiple barriers to successful clinical implementation of AI models, with most remaining in the research phase (41). In addition to an appropriate and well-curated training set and robust clinical validation, optimization of clinical workflows and interpretation of the results in the context of other clinical information is necessary (41, 42). Accordingly, AI tools can be developed to support and augment the expert opinion of pathologists and physicians—an idea embodied in the “physician-in-the-loop” paradigm (43–45).

We previously developed the Genomic Probability Score AI (GPSai™) model (36), which is integrated into the routine tumor profiling workflow and uses genomic and transcriptomic data to predict tissue of origin. The most recent version of this tool, presented in this study, was developed through deep learning training of a curated dataset of 201,612 cases and predicts histologic diagnoses with greatly enhanced granularity and performance compared with other commercially available tests. In addition to validating the accuracy and performance of GPSai, we addressed the hypothesis that this tool would have clinical impact by driving changes in diagnosis and altering guideline-directed targeted therapy eligibility in a meaningful subset of patients. Together, this study shows that GPSai demonstrates high accuracy and potential clinical relevance, supporting a “physician-in-the-loop” approach to the diagnosis of CUP and other pathologically ambiguous tumors.

## Materials and Methods

### Specimen selection

All research-eligible historic cases in the Caris database from the years 2019 to 2023 were used to train the model (*N* = 201,612) and for retrospective validation (*N* = 21,549, including 443 CUP). Cases were selected contingent on having (i) both gene expression and DNA variant data; (ii) an International Classification of Disease primary tumor site; and (iii) a histology that mapped to one of 90 OncoTree (RRID: SCR_026218)-based labels (46) reportable by the model (Supplementary Fig. S1). Baseline demographic information is shown in Supplementary Fig. S2 and was comparable between training, validation, and CUP cohorts. Data from cases profiled between March and October 2024 after the official launch of the GPSai deep learning model were also analyzed (*N* = 80,308). Of this cohort, 72,421 non-CUP cases and 3,850 CUP cases were used for prospective validation.

### RNA and DNA sequencing

Molecular profiling was performed at Caris Life Sciences, a College of American Pathologists/Clinical Laboratory Improvement Amendments–certified laboratory with additional ISO15189 and ISO13485 certifications. Because this study utilized samples spanning 6 years, the methods (including materials and software) used for DNA and RNA profiling evolved over time. Initially, DNA was profiled using a 592 whole-gene panel prior to adoption of whole exome sequencing (WES) with targeted enrichment of 720 clinically relevant genes in 2020; whole transcriptome sequencing (WTS) was performed separately. In 2023, this approach was replaced by MI Tumor Seek Hybrid™, which analyzes RNA and DNA from the same total nucleic acid extraction. In summary, formalin-fixed, paraffin-embedded slides underwent review by a board-certified pathologist to measure tumor content and mark the area for microdissection. A minimum of 20% tumor content in the area for microdissection was required for NGS. A range of tumor content levels (20%–100%) were included in the training and validation cohorts. Nucleic acid was extracted using appropriate formalin-fixed, paraffin-embedded kits for RNA, DNA, or total nucleic acid. DNA sequencing was performed on the NextSeq platform (592 whole-gene panel) or on the NovaSeq 6000 platform (WES; Illumina, Inc.; RRID: SCR_016387). WTS was performed using the Illumina Novaseq 6000 platform to an average of 60M reads. Raw WTS data were demultiplexed, trimmed, counted, and aligned to the human reference genome (hg19 or hg38) by STAR aligner (RRID: SCR_004463; ref. 47). Transcripts per million (TPM) molecules were generated using the Salmon expression pipeline (RRID: SCR_017036; ref. 48). For MI Tumor Seek Hybrid, RNA was labeled during first-strand cDNA synthesis by adapter sequences on the 5′ end of the cDNA primers and NGS was performed (720 clinically relevant genes were sequenced at high depth). Sequencing data were extracted into split FASTQ files (RNA and DNA) for processing. Variants detected were mapped to reference genome hg19 (592 whole-gene panel) or hg38 (WES/Hybrid) using the Burrows–Wheeler Aligner (version 0.7.17; RRID: SCR_010910) embedded in the analysis tools licensed from Sentieon. Bioinformatic tools, including SamTools (RRID: SCR_002105), Pindel (RRID: SCR_000560), and snpEff (RRID: SCR_005191), were incorporated to perform variant calling functions, and annotations were standardized to the Human Genome Variation Society format. Filtering was performed to remove benign variants, low-quality and low-depth variants, or variants determined to be unreliable in several analyses like strand bias and repeat analysis. A threshold of 5% variant allele frequency and five alignments supporting a variant were required to call positive variants. Genetic variants identified were interpreted by board-certified molecular geneticists and categorized as “pathogenic,” “likely pathogenic,” “variant of unknown significance,” “likely benign,” or “benign,” according to the American College of Medical Genetics and Genomics standards. Pathogenic and likely pathogenic variants are counted as “reportable.”

### Deep learning model development

The model was trained in PyTorch (version 2.4.1; RRID: SCR_018536) using variant data, gene expression, and binary sex (male/female). Pathogenic/likely pathogenic somatic variant calls were one-hot encoded at the gene level for 227 clinically relevant genes. Variant frequencies were not included as a feature, and no variant frequencies were filtered beyond the variant frequency filter (>5%) otherwise required for clinical reporting. Approximately 10k gene level TPM features derived from bulk RNA sequencing (RNA-seq) were used in the models. Prior to model training, the TPM data underwent platform dependent normalization to harmonize expression across sequencing platforms.

The pathologic diagnosis entered by the submitting site was mapped to an OncoTree-derived code and used as the training label. Twenty distinct neural networks were trained using training sets consisting of samples from each cancer category that summed to 201,612 total samples (Supplementary Fig. S1), with each neural network corresponding to a branch of the hierarchy. Neural networks were built from the MultilabelClassifier class provided by the PyTorch package. Each network consisted of four fully connected/dense layers, utilizing batch normalization and dropout. Hidden layers were bottlenecked to dimension 256 with the final output layer dimensionality equal to the number of labels that the network was tasked to differentiate. Models were trained using the CrossEntropyLoss loss function, with learning rate = 0.005 and dropout rate = 0.25.

Model optimization was performed using a subset of 46,708 samples. A holdout set consisting of ∼10% of available tumors in each cancer category in the Caris database was used for the retrospective validation (21,106 total mapped tumor samples and 443 CUP). Because the main sequencing platform was changing to MI Tumor Seek Hybrid, only cases run on MI Tumor Seek Hybrid were used for validation purposes. The first network differentiated between 26 major cancer categories, and subsequent networks differentiated between 64 subcategories, which are arranged in a hierarchical manner (Supplementary Fig. S1). Given the hierarchical nature of the diagnostic labels, hierarchical metrics were used in the validations performed (49), as described in Supplementary Fig. S3. Hierarchical positive predictive value (PPV) reports the accuracy of the model in calling all cancer categories. We also report PPV for the top one major cancer category and top two major cancer categories predicted by the model. Metrics reported refer to cases that were given a call by GPSai and do not include cases in which a call was unable to be made.

### Scoring by GPSai

The model assigns a nonnegative score to each category so that the scores of all major categories sum to unity (1.0, or 100%). Subcategory scores also sum to their major categories’ score, as shown in Supplementary Fig. S4. When making a prediction on a novel specimen, the features for that specimen were input through all 20 previously trained neural networks, spanning all major and all subtype disease groups across the labeling hierarchy. Because individual network models were designed to output scores that sum to 1.0, mode scores were adjusted to ensure that subtype probabilities were less-than-or-equal to the probability of the parent type. This was done by iterating through the label hierarchy and, for each label with subtype labels, multiplying each model score of the subtype labels by the corresponding parent model score. This has the effect of (i) ensuring the model scores of all terminal nodes/labels in the label hierarchy sum to 1.0, (ii) ensuring models scores for a set of subtypes always sum to the model score of the corresponding parent label, and (iii) allowing dynamic label prediction/confidence measures across the hierarchy by thresholding on model score.

Call rate was plotted against hierarchical PPV for metastatic cases to identify the optimal threshold score that balanced call rate with PPV (Supplementary Fig. S5). Cases with GPSai results exceeding this threshold score (≥0.55) have the GPSai result included on the final molecular report (this threshold also defines the call rates for validations reported herein). Higher scores suggest additional confidence in the classification. Given that major category scores must sum to unity, only one category can achieve a score of ≥0.55.

### Pathology procedures for review of GPSai results

A “critical value discrepancy” is opened when GPSai assigns a label to a CUP case or if a non-CUP case has a GPSai result that differs from the submitted diagnosis. The threshold score for opening a “critical value discrepancy” is approximately 90% or greater, but the exact threshold is at the discretion of the American Board of Pathology–certified reviewing pathologist. The clinical report is usually only updated (diagnosis, therapeutic biomarker panel, guideline-driven drug associations, and clinical trials) when a CUP case has a GPSai score ≥90% and the call is provable by orthogonal evidence, which includes imaging, diagnostic IHC, hallmark fusions, pathogens, and Catalog of Somatic Mutations in Cancer (COSMIC; RRID: SCR_002260) mutational signatures (e.g., ultraviolet (UV) and tobacco signatures; ref. 50). Of note, none of these orthogonal information sources were directly or indirectly included as input features in the GPSai model. When the GPSai score is ≥90% and results cannot be proven via orthogonal methods, or when there is more than one GPSai result with none ≥90%, the result is included in the clinical report and the option is provided for lineage change at the behest of the ordering physician. Sometimes, a case may have a lower GPSai score, but orthogonal evidence convincingly supports the diagnosis. For non-CUP cases with “critical value discrepancies”, a similar procedure is followed. If the submitted diagnosis has a GPSai score of 0% or if a single GPSai category has a score ≥90%, confirmatory testing is ordered. Otherwise, the result is reported with discretionary lineage change. Criteria for inclusion of a GPSai result on the clinical report are summarized in Supplementary Table S1.

### The Cancer Genome Atlas

RNA-seq data were downloaded for open-access cases directly from The Cancer Genome Atlas (TCGA; RRID: SCR_003193) Genomic Data Commons portal. Pathogenic variant information was obtained from the supplemental section of Sanchez-Vega and colleagues (51).

### Physician surveys

Between March and October 2024, 957 physicians who received a “critical value discrepancy” on the clinical report for their patient (even if this did not result in a diagnosis change) were eligible for survey participation. Molecular Science Liaisons collected 97 survey responses (∼10% response rate). A majority of the respondents were oncologists/treating physicians, and three were pathologists. There were two physicians who submitted two responses each corresponding to two unique patients, which are included as separate responses in the survey results.

### Ethics statement

This study was conducted in accordance with guidelines of the Declaration of Helsinki, Belmont Report, and US Common Rule. This study was Institutional Review Board–approved with waivers of patient written informed consent by the WIRB-Copernicus Group Institutional Review Board (protocols #20242257 and 20244464).

### Data availability

Raw sequencing data are not publicly available due to data size and patient privacy. Summarized GPSai results, relevant pathogenic and likely pathogenic variant data, and a subset of raw sequencing data will be made available upon reasonable request through a letter of intent to Caris Life Sciences (https://www.carislifesciences.com/letter-of-intent/). Please contact the corresponding author for further information.

The GPSai code is included in a zip file within the Supplementary Materials of this article.

## Results

### Accuracy and robustness of GPSai for identifying tumor tissue of origin

**Figure 1:**
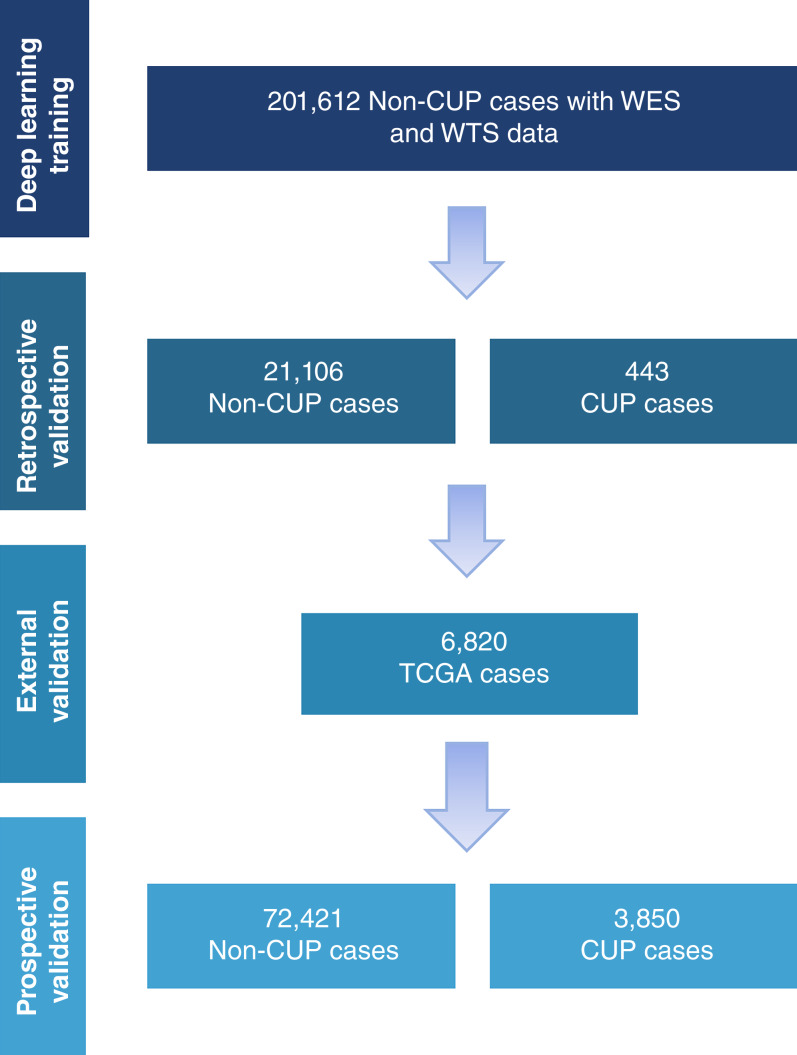
Specimen selection for GPSai model training and validation. A total of 201,612 non-CUP specimens were used to train the model, 21,106 non-CUP specimens and 443 CUP specimens were used for retrospective validation, 6,820 TCGA cases were used for external validation, and 72,421 non-CUP and 3,850 CUP specimens were used for prospective validation, respectively.

The overall scheme for the development and retrospective validation of GPSai is shown in Fig. 1. We first determined the percentage of CUP and non-CUP cases that GPSai was able to classify into one of 90 categories/subcategories reportable by the model, revealing a call rate of 84.0% (*n* = 372/443) in CUP cases and 96.3% (*n* = 20,325/21,106) in non-CUP cases. We then evaluated the accuracy of GPSai in the non-CUP cases compared with the pathologist-submitted diagnostic labels for cases in which GPSai was able to make a call. The global accuracy for the top major cancer category predicted by the model was 95.0%, which was further improved to 98.3% when considering the top two major categories predicted by the model. Hierarchical PPV, which incorporates accuracy of the cancer subcategories, was 93.3%. Performance of the model was only modestly reduced in samples from metastatic sites compared with primary sites (Table 1). Hierarchical PPV and hierarchical sensitivity exceeded 91% for specific metastatic sites (liver, lung, lymph node, bone, and brain; Supplementary Table S2).

**Table 1:** GPSai model performance in retrospective validation

​	Samples, *N*	Call rate, %	hPPV^a^, %	hSens^a^, %	TOP1 PPV, %	TOP2 PPV, %
Non-CUP	​	​	​	​	​	​
Global	21,106	96.3	93.3	92.7	95.0	98.3
Primary	12,668	97.2	94.4	93.7	96.3	98.9
Metastatic	8,438	94.8	91.8	91.1	93.2	97.4
CUP	443	84.0	N/A	N/A	N/A	N/A

Abbreviations: hPPV, hierarchical PPV; hSens, hierarchical sensitivity.

a Hierarchical metrics are reported because of the hierarchical nature of the diagnostic labels (Supplementary Fig. S1). A description of how the hierarchical metrics were calculated is shown in Supplementary Fig. S3. TOP1 = top one major category selected by the GPSai model. TOP2 = top two major categories selected by the GPSai model. PPV is reported for TOP1 and TOP2.

**Figure 2:**
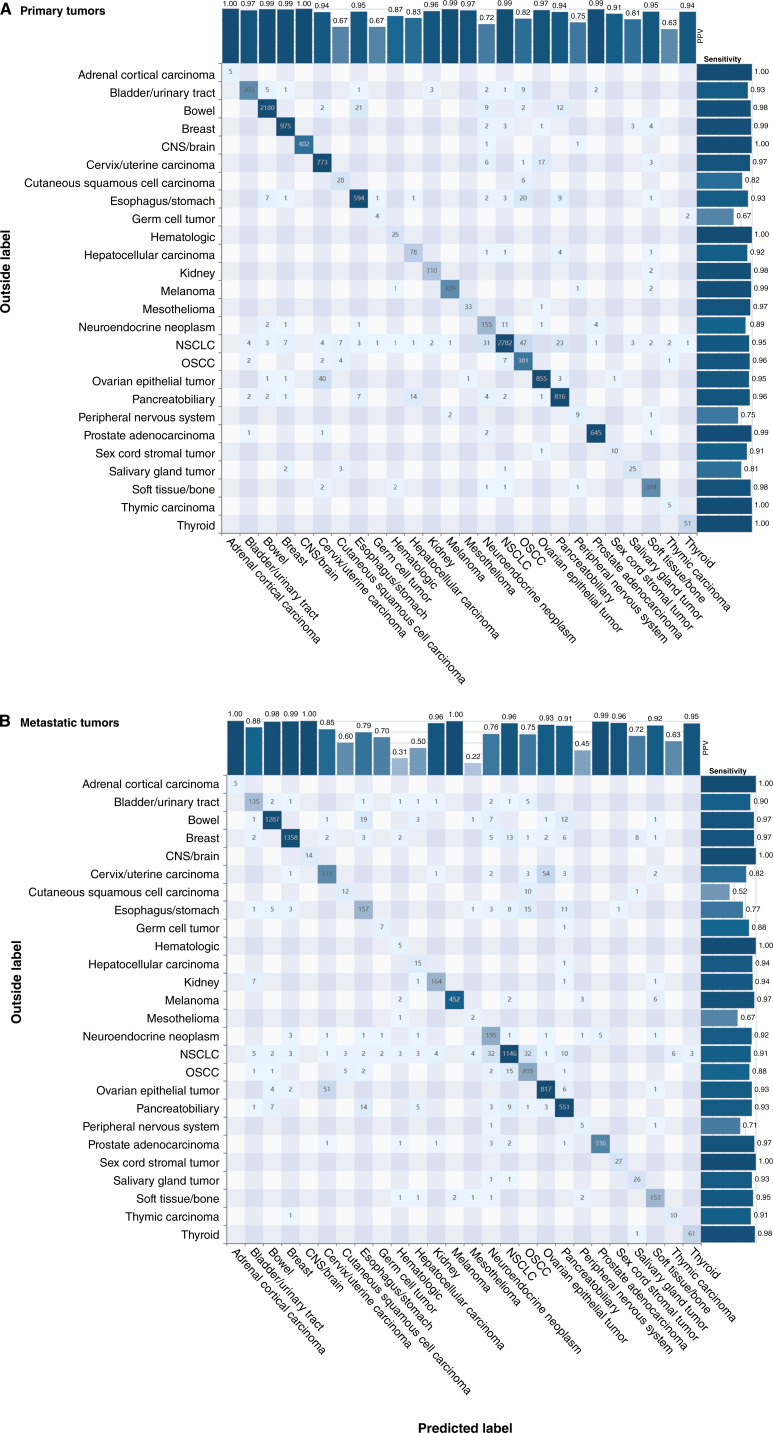
Retrospective validation of GPSai. Model performance per major cancer category is shown for primary (**A**) and metastatic (**B**) tumors. Confusion matrices show numbers of each predicted tumor type in the boxes, with numbers of correctly predicted tumors (matching submitted diagnosis) in each category shown in the diagonal. PPV for each category is indicated by the bars on top, and sensitivity by the bars to the right. Prospective validation results are shown in Supplementary Fig. S8 and Supplementary Table S3. CNS, central nervous system.

We next evaluated how well GPSai performed for each individual cancer category. As seen in the global validation, its performance for individual cancer categories was only slightly reduced for samples from metastatic sites compared with primary sites (Fig. 2A and B). The model was able to accurately classify commonly diagnosed tumor types [e.g., non–small cell lung cancer (NSCLC), breast cancer, bowel cancer, ovarian epithelial cancer, pancreatobiliary cancer, prostate cancer, melanoma, cervix/uterine cancer, and stomach/esophagus] with high accuracy (PPV) and sensitivity in both primary and metastatic tumors; many of these tumor types achieved 98% to 99% PPV and sensitivity. Some miscalls were observed for metastatic cervical/uterine tumors (85% PPV), which were primarily classified as ovarian/fallopian/peritoneal tumors. Miscalls for stomach/esophagus (79% PPV) were more dispersed, but orogenital squamous cell carcinoma (OSCC) and pancreatobiliary were the most common incorrectly predicted categories (Fig. 2A and B). Model performance for all 90 cancer categories is shown in Supplementary Figs. S6 and S7.

### External TCGA validation

**Figure 3:**
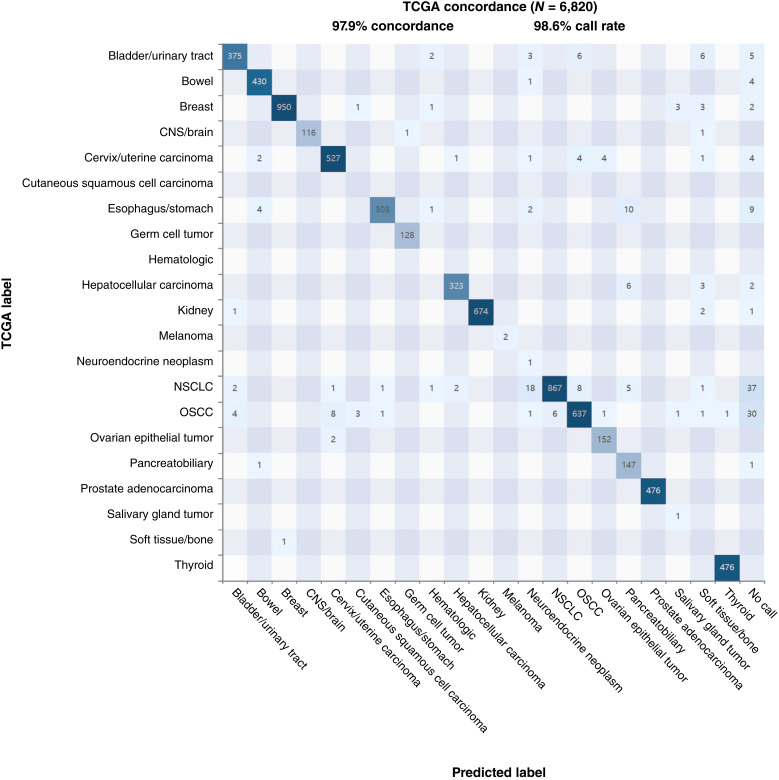
Validation of GPSai in the TCGA dataset. The confusion matrix shows GPSai concordance for each cancer category among 6,820 TCGA samples. Numbers of each predicted tumor type are shown in the boxes, with numbers of correctly predicted tumors (matching TCGA label) in each category shown in the diagonal. CNS, central nervous system.

As an external validation, we applied the model to TCGA data from 6,820 primary and metastatic tumors (Fig. 3). We first aligned the 26 broad cancer categories predicted by our model with TCGA-designated tumor types. The external validation showed 97.9% overall concordance and a 98.6% call rate for GPSai. Bowel, breast, cervix/uterine, kidney, NSCLC, OSCC, prostate, and thyroid cancers were the most represented categories in the TCGA dataset. Only NSCLC demonstrated a cluster of miscalls in neuroendocrine (*n* = 18/943) and OSCC (*n* = 8/943) categories, illustrating some ambiguity between these three cancer types. A majority of “no calls” were also in NSCLC (*n* = 37/943) and OSCC (*n* = 30/694) categories (Fig. 3).

### Prospective validation and quantification of diagnosis changes prompted by GPSai

**Figure 4:**
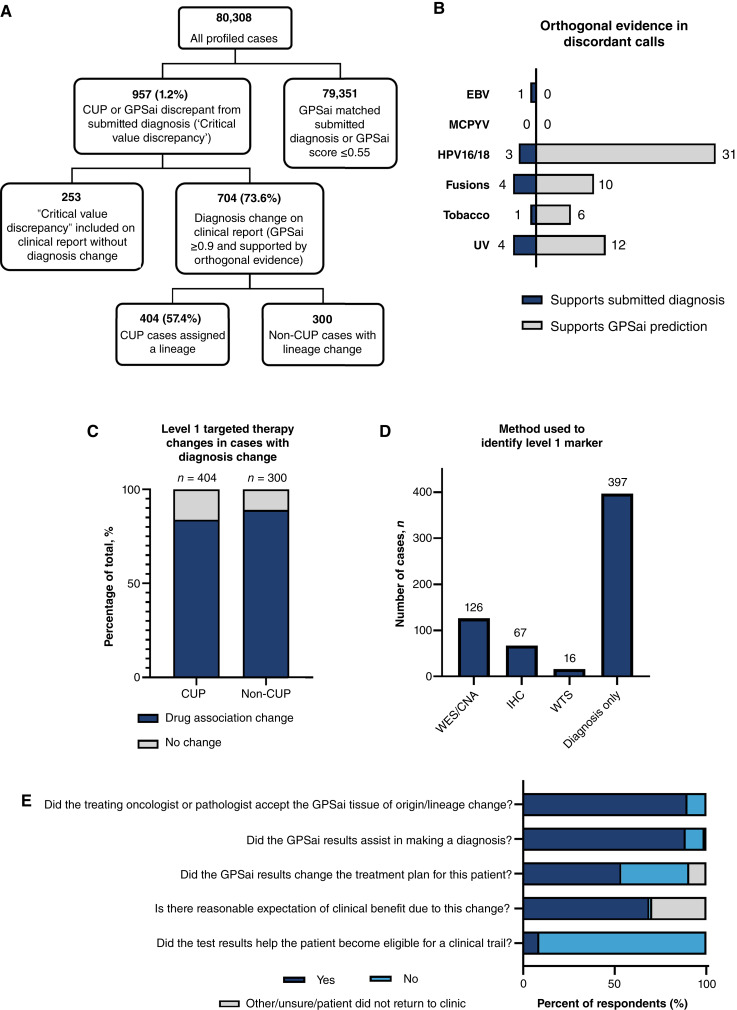
Clinical relevance of GPSai. **A–D,** Quantification of diagnosis changes and level 1 targeted therapy recommendation changes prompted by GPSai. **A,** Cases with “critical value discrepancies” and diagnosis changes supported by orthogonal evidence out of all profiled cases between March and October, 2024. **B,** Cases in which the GPSai call was confirmed by select orthogonal methods are shown. **C,** Percentage of cases with changes in level 1 targeted therapy recommendations among all cases with diagnosis change prompted by GPSai. Although some cases were eligible/ineligible for more than one drug, no more than one drug per case was included in this analysis. **D,** Breakdown of the technologies used to identify the biomarker for level 1 targeted therapy treatment associations. If drug eligibility could be driven by WES/copy-number alterations (CNA) or WTS, that drug was chosen first, followed by IHC-driven drugs. If no biomarker-driven drug association changes were present, diagnosis-only changes were recorded. **E,** Physicians who received a GPSai “critical value discrepancy” on the clinical report for their patient were surveyed to understand how the result affected patient care. There were 97 survey responses corresponding to 97 unique patients.

To gain a better understanding of the clinical relevance of GPSai, we next explored its implementation in the course of routine molecular testing conducted over 8 months. We validated the performance of GPSai in 72,421 non-CUP cases that had tumor type labels that could be mapped to a GPSai label and in 3,850 CUP cases. This prospective validation yielded 95.4% overall accuracy in non-CUP cases and a 82.1% CUP call rate (*n* = 3,159/3,850), closely matching our retrospective validation (Supplementary Fig. S8; Supplementary Table S3). Of 80,308 total profiled cases over the eight-month period, 1.2% (*n* = 957) had a “critical value discrepancy” from the submitted diagnosis (these are opened at the pathologists’ discretion when the GPSai score is approximately 90% or greater) (Fig. 4A). Of cases with a “critical value discrepancy,” the diagnosis was ultimately changed in 73.6% (*n* = 704), representing 0.88% of all profiled cases. Importantly, without detailed case-by-case review, it is not possible to determine the reason that the other 253 cases did not undergo an official diagnosis change; this could be related to lack of corroborating evidence or alternatively that the physician treated according to the GPSai diagnosis but did not request an official diagnosis change. In addition, we cannot speculate on the ultimate diagnoses of cases that had GPSai calls without a “critical value discrepancy” opened.

We next examined the breakdown of cases with a diagnosis change. A small majority (57.4%; *n* = 404) of cases with diagnosis change were submitted as CUP, and 42.6% (*n* = 300) were non-CUP cases that could be considered pathologically ambiguous misdiagnoses (Fig. 4A; Supplementary Table S4). As described in “Materials and Methods,” in order for a “critical value discrepancy” to qualify for diagnosis change on the clinical report, the GPSai score generally must be ≥90% and supported by confirmatory data, which could include diagnostic IHC, COSMIC mutational signatures [e.g., UV and tobacco (50)], hallmark fusions (e.g., *TMPRSS2:ERG*), viral reads (EBV, MCPYV, and HPV16/18), imaging, or clinical history. We specifically examined viral reads, UV and tobacco mutational signatures, and fusions among cases with a discrepant GPSai call from the outside diagnosis, which revealed that the orthogonal evidence more often supported GPSai calls over the outside diagnosis (Fig. 4B; Supplementary Table S5). This analysis underscores the utility of GPSai to act as a “second opinion” for CUP and other pathologically ambiguous metastatic tumors submitted for molecular testing, thus prompting additional workup that should be considered in conjunction with the GPSai result to achieve an accurate diagnosis.

### Quantification of level 1 targeted therapy recommendation changes suggested by GPSai

Cases that had diagnosis changes were also analyzed for level 1 drug association changes. As actual treatment data for these cases were not available, level 1 changes were defined solely by the prescribing information on the drug’s label. National Comprehensive Cancer Network guideline–directed therapies not represented on the drug’s label were not considered. A case was categorized as having a level 1 drug association change if the case became eligible for, or ineligible for, a targeted therapy due to the diagnosis change. In practice, physicians may have been treating some CUP cases with these drugs already, as they may have suspected the GPSai-derived tumor type prior to testing.

Over 8 months of testing, GPSai suggested drug association changes in 83.9% of CUP (*n* = 339) and 89.0% (*n* = 267) of non-CUP cases (Fig. 4C). Whereas all of these changes were rooted in tumor type changes, 34.5% (*n* = 209) were also driven by the presence of a companion diagnostic biomarker (Fig. 4D). A full list of therapeutic eligibility and ineligibility associated with these lineage changes is shown in Supplementary Fig. S9. Changes to eligibility for immunotherapies, such as pembrolizumab, were the most common, due to the large percentage of cases with diagnosis changes from or to NSCLC (Supplementary Table S4). Of the cases with level 1, biomarker-driven changes, we determined that biomarkers were identified by WES in 21% of cases, by WTS in 3% of cases, and by IHC in 11% of cases (Fig. 4D). Together, these results indicate the utility of combining GPSai results with comprehensive molecular profiling to provide an accurate diagnosis and identify lineage-matched, treatment-associated biomarkers.

### Physician survey results

Physicians who received a “critical value discrepancy” on the clinical report for their patient (*n* = 957) were given the opportunity to provide feedback on how GPSai results affected clinical care (Fig. 4E; Supplementary Table S6). Of 97 responses, 89.7% (*n* = 87/97) indicated acceptance of the GPSai results, 88.7% (*n* = 86/97) indicated that GPSai assisted in making a diagnosis, 53.6% (*n* = 52/97) stated that the result changed the treatment plan for their patient, and another 9.3% (*n* = 9/97) were either unsure or indicated that the patient did not return to the clinic. Of the 61 responses that indicated a change or possible change in treatment plan, 68.9% (*n* = 42/61) felt that there was a reasonable expectation of clinical benefit due to the change in treatment. Moreover, 8.9% (*n* = 8/90) of patients became eligible for a clinical trial based on the change in diagnosis.

## Discussion

Although the past two decades have witnessed a surge of diagnostic AI approaches, GPSai is one of a few commercially available tests for tissue of origin identification (18, 21, 25, 36, 52). This tool demonstrates both high accuracy (95.0%) and ability to classify CUP cases (84.0% call rate) in real-world validation cohorts and performs well in metastatic tissue. The large, curated training set used provides excellent coverage of the 90 OncoTree-derived cancer categories predicted by the model, demonstrating superior granularity through prediction of histologic diagnoses, including hematologic malignancies, multiple sarcoma malignancies, neuroendocrine tumors, and soft tissue/bone tumors. Whereas this newly trained model is not directly comparable with our previously published GPSai model (36), it nonetheless represents a significant enhancement in the call rate of CUP cases and the number of cancer categories called. It also offers an entirely unique deep learning approach to classifying tumors with unclear or discrepant primary site diagnoses. Furthermore, our 97.9% accuracy in TCGA samples compares favorably with other models: Tempus TO demonstrated 84.3% accuracy in TCGA samples (21) and CUP-AI-Dx demonstrated 96.7% accuracy in a subcohort of 394 metastatic TCGA samples (24).

Rates of diagnostic discrepancies in anatomic pathology vary between studies and can be difficult to interpret due to biases in cases selected for second opinion review and differences in the types of errors investigated (9–17). Many studies suggest that diagnostic discrepancies are relatively infrequent, but rare tumors and certain tumor sites such as the head and neck may be particularly prone to errors (15, 16). Discrepancies can stem from various factors and have differing effects on clinical management, ranging from minimal impact on patient care to changes in therapeutic modality due to the differential diagnosis (10, 12, 15, 17). Our study differs from other analyses in that we analyzed a biased cohort of tumors submitted for molecular profiling and examined a single type of discrepancy—primary tumor site diagnosis; however, the low rate of potential misdiagnosis that we observed (<1%) is consistent with prior reports. Whereas this rate of misdiagnosis is small, it nonetheless represents a meaningful subset of patients who have the potential to receive inaccurately guided care.

**Figure 5:**
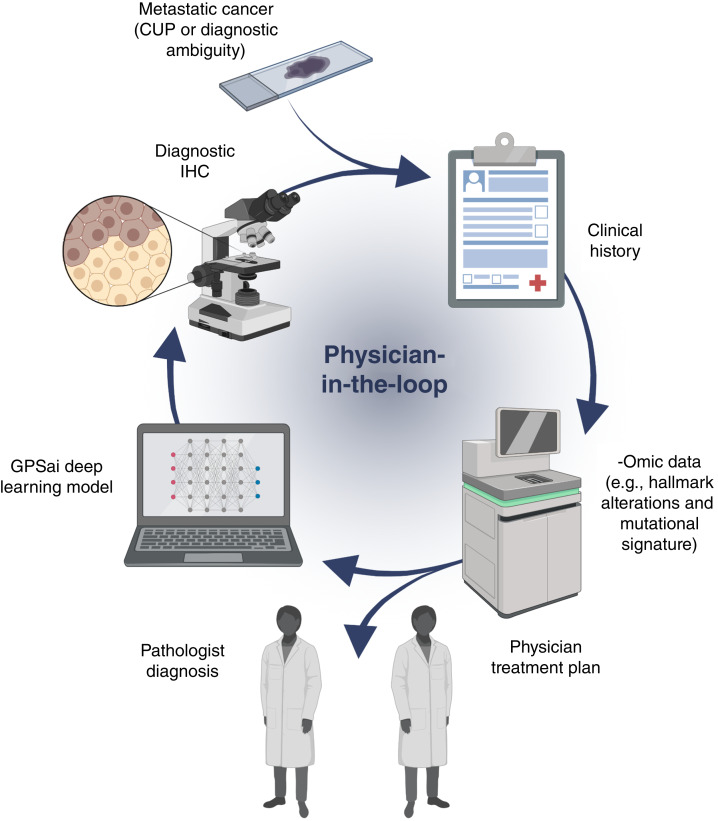
GPSai as part of physician-in-the-loop paradigm for management of CUP and diagnostically ambiguous metastatic tumors. [Created in BioRender. Ribeiro, J. (2025) https://BioRender.com/d83h321.]

As an AI tool, GPSai is relevant to the physician-in-the-loop paradigm that integrates data-driven medical science with physician expertise (43), as illustrated in the schematic in Fig. 5. Whereas a discrepant GPSai label is not sufficient alone to definitively classify a CUP case or support a diagnosis change, integration of GPSai into a comprehensive diagnostic workflow can both aid interpretation of available information and guide further testing to achieve an accurate diagnosis. In cases in which there remains some ambiguity as to the tissue of origin, pertinent information is provided on the clinical report for the physician to assess in order to reach a most probable diagnosis and define the best treatment plan for their patient. Whereas tissue of origin testing is not typically routine or necessary for most patients, the integration of GPSai into the routine profiling workflow increases its clinical applications to diagnosis of patients with CUP (who would typically have an order for GPSai to be run) and also allows unbiased identification of patients with non-CUP tumors that may be incorrectly diagnosed.

Simultaneous biomarker profiling with GPSai also reduces overall tissue requirements, which can be a roadblock to testing, especially for CUP (39, 40). Furthermore, profiling provides additional information to support appropriate treatment planning for patients with CUP or other diagnostically ambiguous tumors. Our study shows that GPSai may affect patient care because of changes in eligibility for FDA-approved therapies or clinical trials. Strictly quantifying level 1 targeted therapy drug associations, we found that a majority of both CUP and non-CUP cases underwent a change in therapeutic eligibility because of the GPSai test result, with a significant subset of these being biomarker-driven. In some cases, this change included the removal of a particular therapy recommendation due to low expectations of success. For instance, we identified nine cases of BRAF/MEK inhibitor (dabrafenib + trametinib) ineligibility due to a diagnosis change to colorectal adenocarcinoma (Supplementary Fig. S9; ref. 53). Physician feedback also indicated a substantial rate of treatment plan change (53.6%) due to GPSai and revealed that eight patients became eligible for a clinical trial, further expanding the potential for GPSai to affect patient treatment options.

Going forward, the goal of GPSai model refinement will be to further improve CUP call rate, expand the number of cancer categories represented by the model, and improve the distinction between certain categories. Whereas molecular-based AI tests such as GPSai are useful in identifying many poorly differentiated tumors that cannot be diagnosed histopathologically, they may nonetheless fail to assign a lineage with high probability in particularly difficult cases. Whereas our cohort lacks reliable grade information, we also postulate that higher tumor grade may contribute to the slightly reduced performance of the model in metastatic tumors compared with primary tumors. A few subcategories also demonstrated lower accuracy in our validation cohorts, which could stem from the rarity of the tumor—and hence, low N for that subcategory—or alternatively, from difficult to distinguish categories or subcategories. Because we observed strong support of orthogonal evidence for GPSai calls, incorporation of this evidence into feature sets used to train the model may also be useful. For example, studies have shown that COSMIC mutational signatures can help distinguish challenging metastatic tumor types, including esophageal squamous cell carcinoma, head and neck squamous cell carcinoma, and OSCC (54–56). Inclusion of COSMIC scores into the GPSai model may prove useful to enhance its ability to call such categories, as esophageal squamous cell carcinoma demonstrated particularly poor PPV (20%) in our study, and the PPV for OSCC was also suboptimal (75%). Regarding cholangiocarcinoma versus pancreatic adenocarcinoma (PDAC), which are also difficult to distinguish pathologically, GPSai was more successful at correctly identifying PDAC (92% PPV), with only 5.7% misidentified as cholangiocarcinoma (*n* = 30/527). Conversely, GPSai misidentified cholangiocarcinoma more frequently (40% PPV), with a higher percentage of cases called as PDAC (11.1%, *n* = 7/63). However, it is important to remember that the pathologist-assigned diagnosis considered as “truth” in this study may be subject to error as well, considering that cholangiocarcinoma and PDAC are in close anatomic proximity and share similar histomolecular features that are difficult to definitively diagnose by IHC (57). We are currently conducting further analyses on the application of GPSai for specific subsets of tumors that are easily misidentified histopathologically, as well as additional validation in an external CUP cohort.

There are a few other limitations to this study. Whereas GPSai is run on all molecularly profiled cases at Caris, nondiagnostic biopsies or biopsies with insufficient tissue available for molecular profiling would still be excluded. Further analysis of the clinical impact of GPSai will also be important going forward. The present study supports its clinical utility by demonstrating changes in diagnosis and subsequent treatment recommendation changes, and physician feedback on implementation of GPSai results further supports its relevance as part of a physician-in-the-loop approach while also providing guidance on areas for improvement. However, the physician survey results must be interpreted with caution because this cohort of respondents was selected based on convenience and is subject to sampling bias, especially given the 10% response rate. Another limitation of our study is that we were unable to directly address the impact of GPSai on clinical outcomes due to inadequate follow-up periods. Whereas site-specific treatment of CUP has led to modestly improved outcomes, particularly among patients with high-accuracy predictions and those with more responsive tumor types (30, 38, 58–60), there is no current consensus on the overall impact of tissue of origin classifiers on patient outcomes (61). In addition, whereas AI tools have the potential to incur significant cost savings through increasing accuracy of diagnosis and treatment and reducing inefficiencies (62), studies to address the economic impact of incorporating GPSai into the routine tumor profiling workflow are also required. Finally, as our study has identified the potential role of this model in aiding challenging diagnoses outside of CUP, optimization of standard operating procedures surrounding the interpretation and validation of diagnosis changes is imperative so as to maximize clinical impact while avoiding unnecessary testing.

In conclusion, this study demonstrates the potential for GPSai to support an AI-based, physician-in-the-loop approach to metastatic cancer diagnostics. Consideration of GPSai results in tandem with other diagnostic tools and biomarker profiling can aid physicians in providing the best care to patients presenting with CUP. Additional benefits may be achieved with the identification of misdiagnoses that could significantly alter care for a small but meaningful subset of patients. Future work will aim to further validate and improve GPSai metrics as well as establish its cost-effectiveness and impact on patient outcomes.

## Supplementary Material

Supplementary Data GPSai code

Supplementary Figure S1 Hierarchy of 90 Oncotree cancer categories covered by GPSai model and sample counts per category used to train the model.

Supplementary Figure S2 Cohort baseline demographics. Age (a) and sex (b) of all patients in training, validation, and CUP cohorts. Gray midline represents the median in (a); median ages of training, validation, and CUP cohorts were 66, 67, and 68, respectively. Kruskal-Wallis test with multiple comparisons (Benjamini and Hochberg) (a) and Chi-square test (b) were used for statistical testing.

Supplementary Figure S3 Calculation of hierarchical positive predictive value (hPPV) and hierarchical sensitivity (hSens). Hierarchical metrics were calculated per sample with values ranging from 0 to 1.0, and then averaged across all samples. The schematic shows level predictions where the “root node” is the broadest category, and not counted toward the calculation. (a) Illustrates 100% hPPV and hSens; (b) illustrates 100% hPPV and 67% hSens; (c) illustrates 67% hPPV and 67% hSens; and (d) illustrates 100% hPPV and 100% hSens, where the model made a more granular prediction than “truth” diagnosis. For example, we commonly receive specimens with a diagnosis of “metastatic carcinoma, consistent with known breast primary” without specifying the subtype. Most likely the cancer was subtyped, but the information wasn’t supplied to us.

Supplementary Figure S4 GPSai model scoring. A representative example of scoring assigned by GPSai is shown. Subcategories (e.g., lung adenocarcinoma, lung squamous cell carcinoma) must sum to unity of the major category score (e.g., non-small cell lung carcinoma).

Supplementary Figure S5 Threshold selection for GPSai. The dashed line at 0.55 indicates the threshold determined to optimize both CUP call rate (green line) and hierarchical positive predictive value (hPPV, blue line) for metastatic cases. A GPS score ≥0.55 indicates high confidence in the tumor type prediction and will be included on the molecular report.

Supplementary Figure S6 Performance per category for all categories (primary tumors). Confusion matrix for primary tumors in retrospective validation cohort. Positive predictive value (PPV) for each category is indicated by the bars above and sensitivity by the bars to the right. Numbers of each predicted tumor type are shown in the boxes, with darker shading of the diagonal indicating a higher number of correctly predicted tumors (matching outside label).

Supplementary Figure S7 Performance per category for all categories (metastatic tumors). Confusion matrix for metastatic tumors in retrospective validation cohort. Positive predictive value (PPV) for each category is indicated by the bars above and sensitivity by the bars to the right. Numbers of each predicted tumor type are shown in the boxes, with darker shading of the diagonal indicating a higher number of correctly predicted tumors (matching outside label).

Supplementary Figure S8 Prospective validation. Model performance per major cancer category is shown for primary and metastatic tumors (N = 72,421). Confusion matrix shows numbers of each predicted tumor type in the boxes, with numbers of correctly predicted tumors (matching submitted diagnosis) in each category shown in the diagonal. Positive predictive value (PPV) for each category is indicated by the bars above and sensitivity by the bars to the right.

Supplementary Figure S9 Changes in drug eligibility based on GPSai results. Based on diagnosis & biomarker or diagnosis-only, all cases with added eligibility (a) or ineligibility (b) for the indicated drugs.

Supplementary Table S1 Pathology procedures for review of GPSai results.

Supplementary Table S2 GPSai model performance in metastatic sites.

Supplementary Table S3 GPSai model performance in prospective validation.

Supplementary Table S4 Cases with diagnosis change due to GPSai results.

Supplementary Table S5 Fusions as orthogonal evidence in cases with ‘Critical Value Discrepancies’.

Supplementary Table S6 Physician survey results.

## References

[1] Massard C , Loriot Y, Fizazi K. Carcinomas of an unknown primary origin—diagnosis and treatment. Nat Rev Clin Oncol 2011;8:701–10. 22048624 10.1038/nrclinonc.2011.158

[2] Rassy E , Pavlidis N. The currently declining incidence of cancer of unknown primary. Cancer Epidemiol 2019;61:139–41. 31254795 10.1016/j.canep.2019.06.006

[3] Mnatsakanyan E , Tung WC, Caine B, Smith-Gagen J. Cancer of unknown primary: time trends in incidence, United States. Cancer Causes Control 2014;25:747–57. 24710663 10.1007/s10552-014-0378-2

[4] American Cancer Society . Key statistics for cancers of unknown primary. 2025 [cited 2025 June]. Available from: https://www.cancer.org/cancer/types/cancer-unknown-primary/about/key-statistics.html.

[5] Huebner G , Link H, Kohne CH, Stahl M, Kretzschmar A, Steinbach S, . Paclitaxel and carboplatin vs gemcitabine and vinorelbine in patients with adeno- or undifferentiated carcinoma of unknown primary: a randomised prospective phase II trial. Br J Cancer 2009;100:44–9. 19066607 10.1038/sj.bjc.6604818 PMC2634671

[6] Hess KR , Abbruzzese MC, Lenzi R, Raber MN, Abbruzzese JL. Classification and regression tree analysis of 1000 consecutive patients with unknown primary carcinoma. Clin Cancer Res 1999;5:3403–10. 10589751

[7] Kaaks R , Sookthai D, Hemminki K, Krämer A, Boeing H, Wirfält E, . Risk factors for cancers of unknown primary site: results from the prospective EPIC cohort. Int J Cancer 2014;135:2475–81. 24692151 10.1002/ijc.28874

[8] Kang S , Jeong JH, Yoon S, Yoo C, Kim KP, Cho H, . Real-world data analysis of patients with cancer of unknown primary. Sci Rep 2021;11:23074. 34845302 10.1038/s41598-021-02543-1 PMC8630084

[9] Peck M , Moffat D, Latham B, Badrick T. Review of diagnostic error in anatomical pathology and the role and value of second opinions in error prevention. J Clin Pathol 2018;71:995–1000. 30068638 10.1136/jclinpath-2018-205226

[10] Tsung JS . Institutional pathology consultation. Am J Surg Pathol 2004;28:399–402. 15104305 10.1097/00000478-200403000-00015

[11] Woolgar JA , Ferlito A, Devaney KO, Rinaldo A, Barnes L. How trustworthy is a diagnosis in head and neck surgical pathology? A consideration of diagnostic discrepancies (errors). Eur Arch Otorhinolaryngol 2011;268:643–51. 21340559 10.1007/s00405-011-1526-x

[12] Sharif MA , Hamdani SNR. Second opinion and discrepancy in the diagnosis of soft tissue lesions at surgical pathology. Indian J Pathol Microbiol 2010;53:460–4. 20699503 10.4103/0377-4929.68277

[13] Grevenkamp F , Kommoss F, Kommoss F, Lax S, Fend F, Wallwiener D, . Second opinion expert pathology in endometrial cancer: potential clinical implications. Int J Gynecol Cancer 2017;27:289–96. 27922981 10.1097/IGC.0000000000000870

[14] Gordetsky J , Collingwood R, Lai WS, Del Carmen Rodriquez Pena M, Rais-Bahrami S. Second opinion expert pathology review in bladder cancer: implications for patient care. Int J Surg Pathol 2018;26:12–7. 28905666 10.1177/1066896917730903

[15] Rakha EA , Adebayo LA, Abbas A, Hodi Z, Lee AHS, Ellis IO. Second opinion (external specialist referral) practice of breast pathology: the Nottingham experience. Histopathology 2023;83:394–405. 37356966 10.1111/his.14993

[16] Kronz JD , Westra WH. The role of second opinion pathology in the management of lesions of the head and neck. Curr Opin Otolaryngol Head Neck Surg 2005;13:81–4. 15761280 10.1097/01.moo.0000156162.20789.66

[17] Johnson SM , Samulski TD, O’Connor SM, Smith SV, Funkhouser WK, Broaddus RR, . Clinical and financial implications of second-opinion surgical pathology review. Am J Clin Pathol 2021;156:559–68. 33769453 10.1093/ajcp/aqaa263

[18] Erlander MG , Ma XJ, Kesty NC, Bao L, Salunga R, Schnabel CA. Performance and clinical evaluation of the 92-gene real-time PCR assay for tumor classification. J Mol Diagn 2011;13:493–503. 21708287 10.1016/j.jmoldx.2011.04.004 PMC3157614

[19] Tothill RW , Shi F, Paiman L, Bedo J, Kowalczyk A, Mileshkin L, . Development and validation of a gene expression tumour classifier for cancer of unknown primary. Pathology 2015;47:7–12. 25485653 10.1097/PAT.0000000000000194

[20] Ma W , Wu H, Chen Y, Xu H, Jiang J, Du B, . New techniques to identify the tissue of origin for cancer of unknown primary in the era of precision medicine: progress and challenges. Brief Bioinform 2024;25:bbae028. 38343328 10.1093/bib/bbae028 PMC10859692

[21] Michuda J , Breschi A, Kapilivsky J, Manghnani K, McCarter C, Hockenberry AJ, . Validation of a transcriptome-based assay for classifying cancers of unknown primary origin. Mol Diagn Ther 2023;27:499–511. 37099070 10.1007/s40291-023-00650-5 PMC10300170

[22] Moiso E , Farahani A, Marble HD, Hendricks A, Mildrum S, Levine S, . Developmental deconvolution for classification of cancer origin. Cancer Discov 2022;12:2566–85. 36041084 10.1158/2159-8290.CD-21-1443 PMC9627133

[23] Vibert J , Pierron G, Benoist C, Gruel N, Guillemot D, Vincent-Salomon A, . Identification of tissue of origin and guided therapeutic applications in cancers of unknown primary using deep learning and RNA sequencing (TransCUPtomics). J Mol Diagn 2021;23:1380–92. 34325056 10.1016/j.jmoldx.2021.07.009

[24] Zhao Y , Pan Z, Namburi S, Pattison A, Posner A, Balachander S, . CUP-AI-Dx: a tool for inferring cancer tissue of origin and molecular subtype using RNA gene-expression data and artificial intelligence. EBioMedicine 2020;61:103030. 33039710 10.1016/j.ebiom.2020.103030 PMC7553237

[25] Pillai R , Deeter R, Rigl CT, Nystrom JS, Miller MH, Buturovic L, . Validation and reproducibility of a microarray-based gene expression test for tumor identification in formalin-fixed, paraffin-embedded specimens. J Mol Diagn 2011;13:48–56. 21227394 10.1016/j.jmoldx.2010.11.001 PMC3070545

[26] Ross JS , Wang K, Gay L, Otto GA, White E, Iwanik K, . Comprehensive genomic profiling of carcinoma of unknown primary site: new routes to targeted therapies. JAMA Oncol 2015;1:40–9. 26182302 10.1001/jamaoncol.2014.216

[27] Penson A , Camacho N, Zheng Y, Varghese AM, Al-Ahmadie H, Razavi P, . Development of genome-derived tumor type prediction to inform clinical cancer care. JAMA Oncol 2020;6:84–91. 31725847 10.1001/jamaoncol.2019.3985 PMC6865333

[28] Marquard AM , Birkbak NJ, Thomas CE, Favero F, Krzystanek M, Lefebvre C, . TumorTracer: a method to identify the tissue of origin from the somatic mutations of a tumor specimen. BMC Med Genomics 2015;8:58. 26429708 10.1186/s12920-015-0130-0 PMC4590711

[29] Jiao W , Atwal G, Polak P, Karlic R, Cuppen E, Danyi A, ; PCAWG Tumor Subtypes and Clinical Translation Working Group. A deep learning system accurately classifies primary and metastatic cancers using passenger mutation patterns. Nat Commun 2020;11:728. 32024849 10.1038/s41467-019-13825-8 PMC7002586

[30] Moon I , LoPiccolo J, Baca SC, Sholl LM, Kehl KL, Hassett MJ, . Machine learning for genetics-based classification and treatment response prediction in cancer of unknown primary. Nat Med 2023;29:2057–67. 37550415 10.1038/s41591-023-02482-6 PMC11484892

[31] He B , Dai C, Lang J, Bing P, Tian G, Wang B, . A machine learning framework to trace tumor tissue-of-origin of 13 types of cancer based on DNA somatic mutation. Biochim Biophys Acta Mol Basis Dis 2020;1866:165916. 32771416 10.1016/j.bbadis.2020.165916

[32] Liu X , Li L, Peng L, Wang B, Lang J, Lu Q, . Predicting cancer tissue-of-origin by a machine learning method using DNA somatic mutation data. Front Genet 2020;11:674. 32760423 10.3389/fgene.2020.00674 PMC7372518

[33] Nguyen L , Van Hoeck A, Cuppen E. Machine learning-based tissue of origin classification for cancer of unknown primary diagnostics using genome-wide mutation features. Nat Commun 2022;13:4013. 35817764 10.1038/s41467-022-31666-w PMC9273599

[34] Liang Y , Wang H, Yang J, Li X, Dai C, Shao P, . A deep learning framework to predict tumor tissue-of-origin based on copy number alteration. Front Bioeng Biotechnol 2020;8:701. 32850687 10.3389/fbioe.2020.00701 PMC7419421

[35] Zhang Y , Feng T, Wang S, Dong R, Yang J, Su J, . A novel XGBoost method to identify cancer tissue-of-origin based on copy number variations. Front Genet 2020;11:585029. 33329723 10.3389/fgene.2020.585029 PMC7716814

[36] Abraham J , Heimberger AB, Marshall J, Heath E, Drabick J, Helmstetter A, . Machine learning analysis using 77,044 genomic and transcriptomic profiles to accurately predict tumor type. Transl Oncol 2021;14:101016. 33465745 10.1016/j.tranon.2021.101016 PMC7815805

[37] Chakravarty D , Johnson A, Sklar J, Lindeman NI, Moore K, Ganesan S, . Somatic genomic testing in patients with metastatic or advanced cancer: ASCO provisional clinical opinion. J Clin Oncol 2022;40:1231–58. 35175857 10.1200/JCO.21.02767

[38] Laprovitera N , Riefolo M, Ambrosini E, Klec C, Pichler M, Ferracin M. Cancer of unknown primary: challenges and progress in clinical management. Cancers (Basel) 2021;13:451. 33504059 10.3390/cancers13030451 PMC7866161

[39] Zaun G , Borchert S, Metzenmacher M, Lueong S, Wiesweg M, Zaun Y, . Comprehensive biomarker diagnostics of unfavorable cancer of unknown primary to identify patients eligible for precision medical therapies. Eur J Cancer 2024;200:113540. 38316065 10.1016/j.ejca.2024.113540

[40] Huey RW , Shah AT, Reddi HV, Dasari P, Topham JT, Hwang H, . Feasibility and value of genomic profiling in cancer of unknown primary: real-world evidence from prospective profiling study. J Natl Cancer Inst 2023;115:994–7. 37202363 10.1093/jnci/djad095 PMC10407690

[41] Bi WL , Hosny A, Schabath MB, Giger ML, Birkbak NJ, Mehrtash A, . Artificial intelligence in cancer imaging: clinical challenges and applications. CA Cancer J Clin 2019;69:127–57. 30720861 10.3322/caac.21552 PMC6403009

[42] Shao J , Feng J, Li J, Liang S, Li W, Wang C. Novel tools for early diagnosis and precision treatment based on artificial intelligence. Chin Med J Pulm Crit Care Med 2023;1:148–60. 39171128 10.1016/j.pccm.2023.05.001 PMC11332840

[43] Stenzinger A , Alber M, Allgäuer M, Jurmeister P, Bockmayr M, Budczies J, . Artificial intelligence and pathology: from principles to practice and future applications in histomorphology and molecular profiling. Semin Cancer Biol 2022;84:129–43. 33631297 10.1016/j.semcancer.2021.02.011

[44] Bigorra L , Larriba I, Gutiérrez-Gallego R. A physician-in-the-loop approach by means of machine learning for the diagnosis of lymphocytosis in the clinical laboratory. Arch Pathol Lab Med 2022;146:1024–31. 34807976 10.5858/arpa.2021-0044-OA

[45] Kieseberg P , Malle B, Frühwirt P, Weippl E, Holzinger A. A tamper-proof audit and control system for the doctor in the loop. Brain Inform 2016;3:269–79. 27747816 10.1007/s40708-016-0046-2 PMC5106408

[46] Kundra R , Zhang H, Sheridan R, Sirintrapun SJ, Wang A, Ochoa A, . OncoTree: a cancer classification system for precision Oncology. JCO Clin Cancer Inform 2021;5:221–30. 33625877 10.1200/CCI.20.00108 PMC8240791

[47] Dobin A , Davis CA, Schlesinger F, Drenkow J, Zaleski C, Jha S, . STAR: ultrafast universal RNA-seq aligner. Bioinformatics 2013;29:15–21. 23104886 10.1093/bioinformatics/bts635 PMC3530905

[48] Patro R , Duggal G, Love MI, Irizarry RA, Kingsford C. Salmon provides fast and bias-aware quantification of transcript expression. Nat Methods 2017;14:417–9. 28263959 10.1038/nmeth.4197 PMC5600148

[49] Rezende PM , Xavier JS, Ascher DB, Fernandes GR, Pires DEV. Evaluating hierarchical machine learning approaches to classify biological databases. Brief Bioinform 2022;23:bbac216. 35724625 10.1093/bib/bbac216 PMC9310517

[50] Alexandrov LB , Kim J, Haradhvala NJ, Huang MN, Tian Ng AW, Wu Y, . The repertoire of mutational signatures in human cancer. Nature 2020;578:94–101. 32025018 10.1038/s41586-020-1943-3 PMC7054213

[51] Sanchez-Vega F , Mina M, Armenia J, Chatila WK, Luna A, La KC, . Oncogenic signaling pathways in the cancer genome Atlas. Cell 2018;173:321–37.e10. 29625050 10.1016/j.cell.2018.03.035 PMC6070353

[52] Meiri E , Mueller WC, Rosenwald S, Zepeniuk M, Klinke E, Edmonston TB, . A second-generation microRNA-based assay for diagnosing tumor tissue origin. Oncologist 2012;17:801–12. 22618571 10.1634/theoncologist.2011-0466 PMC3380879

[53] Corcoran RB , Atreya CE, Falchook GS, Kwak EL, Ryan DP, Bendell JC, . Combined BRAF and MEK inhibition with dabrafenib and trametinib in BRAF V600-mutant colorectal cancer. J Clin Oncol 2015;33:4023–31. 26392102 10.1200/JCO.2015.63.2471 PMC4669588

[54] Gupta R , Strbenac D, Satgunaseelan L, Cheung VK, Narayanappa H, Ashford B, . Comparing genomic landscapes of oral and cutaneous squamous cell carcinoma of the head and neck: quest for novel diagnostic markers. Mod Pathol 2023;36:100190. 37080394 10.1016/j.modpat.2023.100190

[55] Dutta M , Nakagawa H, Kato H, Maejima K, Sasagawa S, Nakano K, . Whole genome sequencing analysis identifies recurrent structural alterations in esophageal squamous cell carcinoma. PeerJ 2020;8:e9294. 32617189 10.7717/peerj.9294 PMC7323713

[56] Li M , Zhang Z, Wang Q, Yi Y, Li B. Integrated cohort of esophageal squamous cell cancer reveals genomic features underlying clinical characteristics. Nat Commun 2022;13:5268. 36071046 10.1038/s41467-022-32962-1 PMC9452532

[57] Gkountakos A , Martelli FM, Silvestris N, Bevere M, De Bellis M, Alaimo L, . Extrahepatic distal cholangiocarcinoma vs. Pancreatic ductal adenocarcinoma: histology and molecular profiling for differential diagnosis and treatment. Cancers (Basel) 2023;15:1454. 36900245 10.3390/cancers15051454 PMC10001378

[58] Hainsworth JD , Rubin MS, Spigel DR, Boccia RV, Raby S, Quinn R, . Molecular gene expression profiling to predict the tissue of origin and direct site-specific therapy in patients with carcinoma of unknown primary site: a prospective trial of the Sarah Cannon research institute. J Clin Oncol 2013;31:217–23. 23032625 10.1200/JCO.2012.43.3755

[59] Ding Y , Jiang J, Xu J, Chen Y, Zheng Y, Jiang W, . Site-specific therapy in cancers of unknown primary site: a systematic review and meta-analysis. ESMO Open 2022;7:100407. 35248824 10.1016/j.esmoop.2022.100407 PMC8897579

[60] Moran S , Martínez-Cardús A, Sayols S, Musulén E, Balañá C, Estival-Gonzalez A, . Epigenetic profiling to classify cancer of unknown primary: a multicentre, retrospective analysis. Lancet Oncol 2016;17:1386–95. 27575023 10.1016/S1470-2045(16)30297-2

[61] National Comprehensive Cancer Network . NCCN clinical practice guidelines in Oncology (NCCN Guidelines®) occult primary (cancer of unknown primary [CUP]) version 2.2025. 2025 [cited 2025 June]. Available from: https://www.nccn.org/professionals/physician_gls/pdf/occult.pdf.

[62] Khanna NN , Maindarkar MA, Viswanathan V, Fernandes JFE, Paul S, Bhagawati M, . Economics of artificial intelligence in healthcare: diagnosis vs. treatment. Healthcare (Basel) 2022;10:2493. 36554017 10.3390/healthcare10122493 PMC9777836

