# Supplementary material for: GPSai: A Clinically Validated AI Tool for Tissue of Origin Prediction during Routine Tumor Profiling
Source: Cancer Res Commun. 2025 Sep 1;5(9):1477–89. doi: 10.1158/2767-9764.CRC-25-0171 (PMC12399951; doi:10.1158/2767-9764.CRC-25-0171)
Supplement: Supplementary Figure S1 — Hierarchy of 90 Oncotree cancer categories covered by GPSai model and sample counts per category used to train the model. [file crc-25-0171_supplementary_figure_s1_suppsf1.pdf]

**Supplementary Figure S1. Hierarchy of 90 Oncotree cancer categories covered by GPSai model and sample counts per category used to train the model.**

```
label                                                                   count    cummulative_count
----------------------------------------------------------------------  -------  -----------------
├── Adrenal Cortical Carcinoma                                              152                152
├── Pancreatobiliary                                                          4              12622
│   ├── Pancreatic Adenocarcinoma                                          8836               8836
│   ├── Cholangiocarcinoma                                                 2620               2620
│   └── Gallbladder Cancer                                                 1162               1162
├── Bladder/Urinary Tract                                                   378               6348
│   ├── Urothelial Carcinoma                                               5848               5848
│   └── Bladder Adenocarcinoma                                              122                122
├── Bowel                                                                  2753              25022
│   ├── Colorectal Adenocarcinoma                                         20551              20551
│   ├── Small Bowel Carcinoma                                               872                872
│   └── Appendiceal Adenocarcinoma                                          846                846
├── Melanoma                                                               6051               6051
├── Breast                                                                10237              17524
│   ├── Breast Invasive Lobular Carcinoma                                   947                947
│   ├── Breast Invasive Ductal Carcinoma                                   6177               6177
│   └── Metaplastic Breast Cancer                                           163                163
├── CNS/Brain                                                                 1               7778
│   ├── Diffuse Glioma                                                     6959               6959
│   └── Meningioma                                                          818                818
├── Esophagus/Stomach                                                       293              10125
│   ├── Esophagogastric Adenocarcinoma                                      274               8681
│   │   ├── Stomach Adenocarcinoma                                         3228               3228
│   │   ├── Esophageal Adenocarcinoma                                      3197               3197
│   │   └── Adenocarcinoma of the Gastroesophageal Junction                1982               1982
│   └── Esophageal Squamous Cell Carcinoma                                 1151               1151
├── Orogenital Squamous Cell Carcinoma                                     8028               8028
├── Salivary Gland Tumor                                                    924                924
├── Kidney                                                                   59               3149
│   ├── Renal Cell Carcinoma                                               2126               3069
│   │   ├── Papillary Renal Cell Carcinoma                                  156                156
│   │   ├── Chromophobe Renal Cell Carcinoma                                 48                 48
│   │   └── Renal Clear Cell Carcinoma                                      739                739
│   └── Wilms Tumor                                                          21                 21
├── Hepatocellular Carcinoma                                               1222               1222
├── Non-Small Cell Lung Carcinoma                                          2887              32109
│   ├── Lung Adenocarcinoma                                               21201              21201
│   └── Lung Squamous Cell Carcinoma                                       8021               8021
├── Ovarian Epithelial Tumor                                               1618              14355
│   ├── Serous Ovarian/Fallopian Tube/Peritoneal                           2701              10428
│   │   ├── High-Grade Serous Ovarian/Fallopian Tube/Peritoneal Cancer     7218               7218
│   │   └── Low-Grade Serous Ovarian/Fallopian Tube/Peritoneal Cancer       509                509
│   ├── Endometrioid Ovarian Cancer                                         752                752
│   ├── Mucinous Ovarian Cancer                                             308                308
│   ├── Clear Cell Ovarian Cancer                                           763                763
│   └── Ovarian Carcinosarcoma/Malignant Mixed Mesodermal Tumor             486                486
├── Germ Cell Tumor                                                         246                246
├── Mesothelioma                                                            501                501
├── Prostate Adenocarcinoma                                                8795               8795
├── Neuroendocrine Neoplasm                                                  47               3913
│   ├── Paraganglioma/Pheochromocytoma                                       66                 66
│   ├── Merkel Cell Carcinoma                                               169                169
│   ├── Well/Moderately-Differentiated Neuroendocrine Tumor                 261                261
│   └── Poorly-Differentiated Neuroendocrine Carcinoma                     1326               3370
│       ├── Small Cell Neuroendocrine Carcinoma                            1722               1722
│       └── Large Cell Neuroendocrine Carcinoma                             322                322
├── Thymic Carcinoma                                                        131                131
├── Thyroid                                                                 480               1827
│   ├── Anaplastic Thyroid Cancer                                           127                127
│   ├── Well-Differentiated Thyroid Cancer                                    6                999
│   │   ├── Papillary Thyroid Cancer                                        861                861
│   │   └── Follicular Thyroid Cancer                                       132                132
│   ├── Hurthle Cell Thyroid Cancer                                          72                 72
│   └── Medullary Thyroid Cancer                                            149                149
├── Cervix/Uterine Carcinoma                                               4201              14605
│   ├── Cervical Adenocarcinoma                                             769                769
│   ├── Uterine Endometrioid Carcinoma                                     4728               4728
│   ├── Uterine Serous Carcinoma/Uterine Papillary Serous Carcinoma        2899               2899
│   ├── Uterine Carcinosarcoma/Uterine Malignant Mixed Mullerian Tumor     1610               1610
│   └── Uterine Clear Cell Carcinoma                                        398                398
├── Soft Tissue/Bone                                                       1939               6593
│   ├── Gastrointestinal Stromal Tumor                                     1361               1361
│   ├── Endometrial Stromal Sarcoma                                         226                226
│   ├── Liposarcoma                                                         510                510
│   ├── Synovial Sarcoma                                                    122                122
│   ├── Leiomyosarcoma                                                     1433               1433
│   ├── Osteosarcoma                                                        201                201
│   ├── Ewing Sarcoma                                                       146                146
│   ├── Chondrosarcoma                                                      175                175
│   ├── Angiosarcoma                                                        287                287
│   └── Rhabdomyosarcoma                                                    193                193
├── Sex Cord Stromal Tumor                                                   45                441
│   ├── Sertoli-Leydig Cell Tumor                                            49                 49
│   └── Granulosa Cell Tumor                                                347                347
├── Cutaneous Squamous Cell Carcinoma                                       703                703
├── Peripheral Nervous System                                                31                299
│   ├── Malignant Peripheral Nerve Sheath Tumor                             107                107
│   ├── Schwannoma                                                           58                 58
│   └── Neuroblastoma                                                       103                103
└── Hematological                                                           550                550
```
