# Supplementary material for: GPSai: A Clinically Validated AI Tool for Tissue of Origin Prediction during Routine Tumor Profiling
Source: Cancer Res Commun. 2025 Sep 1;5(9):1477–89. doi: 10.1158/2767-9764.CRC-25-0171 (PMC12399951; doi:10.1158/2767-9764.CRC-25-0171)

Supplementary Figure S2. Cohort baseline demographics. Age **(A)** and sex **(B)** of all patients in training, validation, and CUP cohorts. Gray midline represents the median in **(A)**; median ages of training, validation, and CUP cohorts were 66, 67, and 68, respectively. Kruskal-Wallis test with multiple comparisons (Benjamini and Hochberg) **(A)** and Chi-square test **(B)** were used for statistical testing.

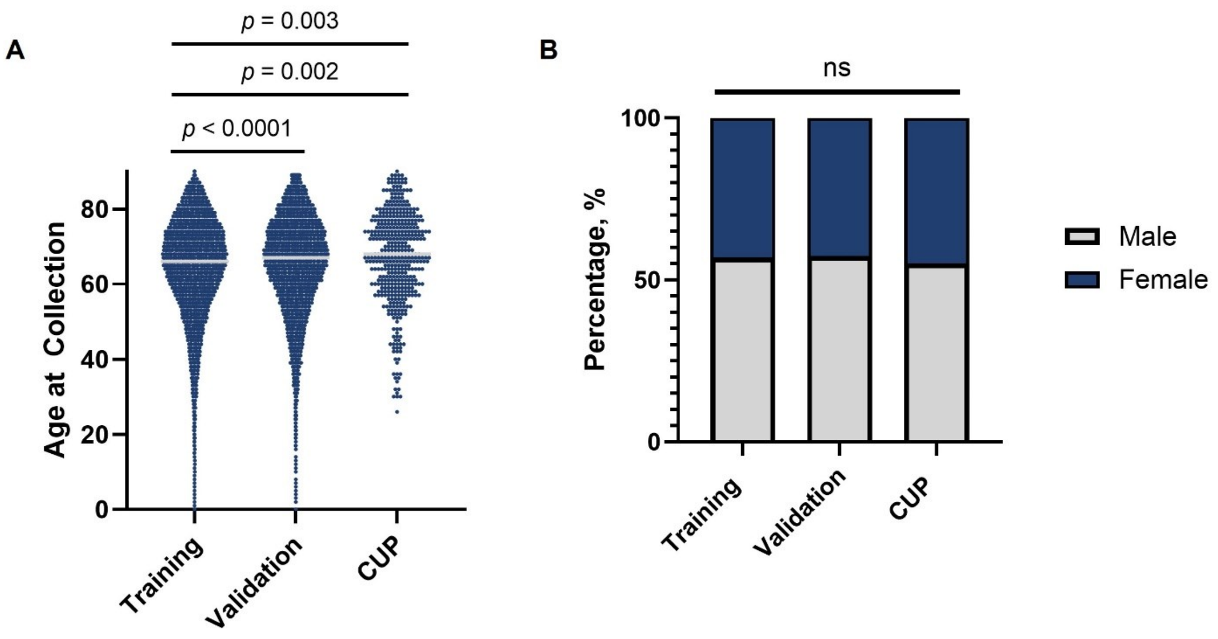

Supplement: Supplementary Figure S2 — Cohort baseline demographics. Age (a) and sex (b) of all patients in training, validation, and CUP cohorts. Gray midline represents the median in (a); median ages of training, validation, and CUP cohorts were 66, 67, and 68, respectively. Kruskal-Wallis test with multiple comparisons (Benjamini and Hochberg) (a) and Chi-square test (b) were used for statistical testing. [file crc-25-0171_supplementary_figure_s2_suppsf2.pdf]
