# Supplementary material for: GPSai: A Clinically Validated AI Tool for Tissue of Origin Prediction during Routine Tumor Profiling
Source: Cancer Res Commun. 2025 Sep 1;5(9):1477–89. doi: 10.1158/2767-9764.CRC-25-0171 (PMC12399951; doi:10.1158/2767-9764.CRC-25-0171)

Supplementary Figure S3. Calculation of hierarchical positive predictive value (hPPV) and hierarchical sensitivity (hSens). Hierarchical metrics were calculated per sample with values ranging from 0-1.0, and then averaged across all samples. The schematic shows level predictions where the "root node" is the broadest category, and not counted toward the calculation. **(a)** Illustrates 100% hPPV and hSens; **(b)** illustrates 100% hPPV and 67% hSens; **(c)** illustrates 67% hPPV and 67% hSens; and **(d)** illustrates 100% hPPV and 100% hSens, where the model made a more granular prediction than "truth" diagnosis. For example, we commonly receive specimens with a diagnosis of "metastatic carcinoma, consistent with known breast primary" without specifying the subtype. Most likely the cancer was subtyped, but the information wasn't supplied to us.

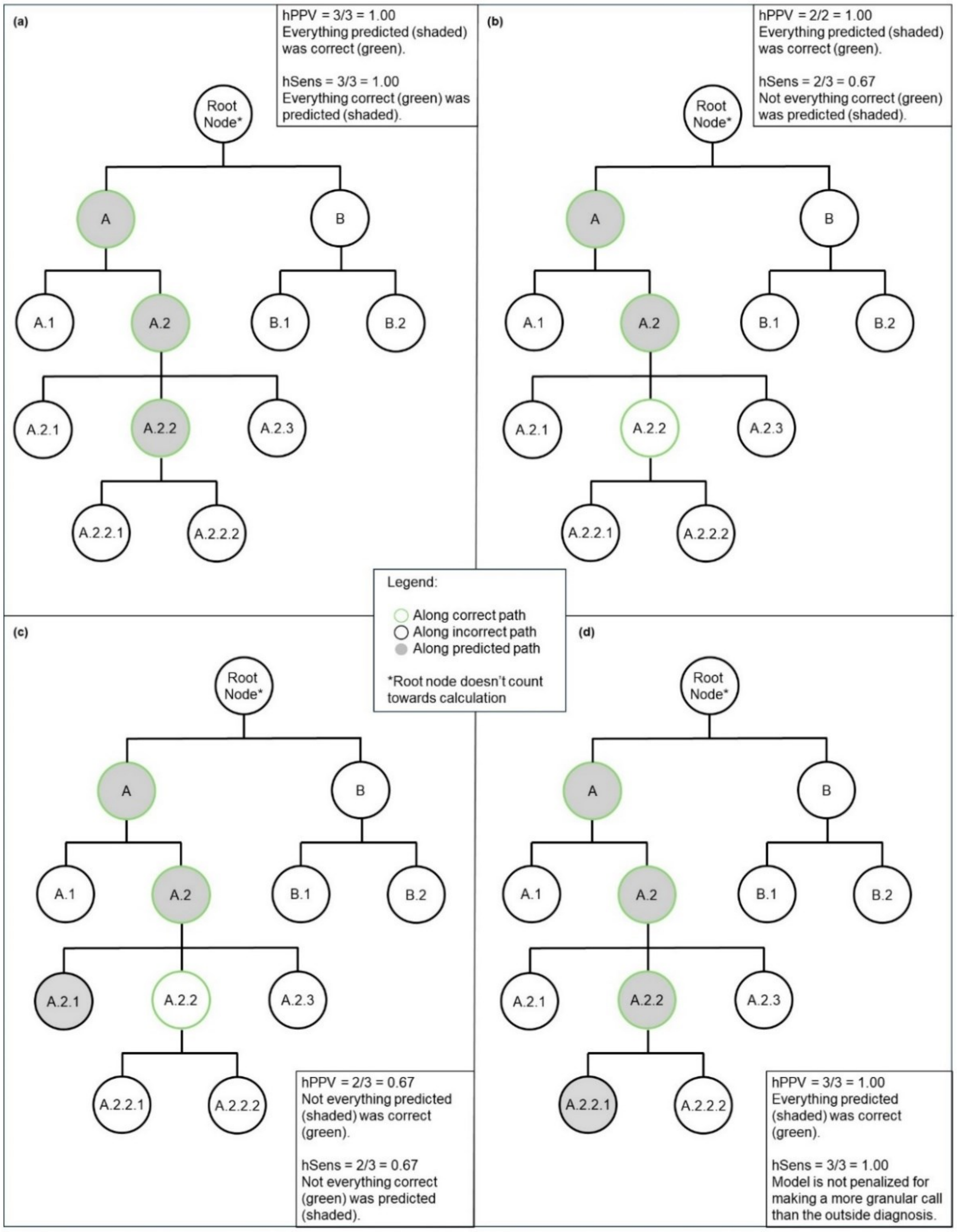

Supplement: Supplementary Figure S3 — Calculation of hierarchical positive predictive value (hPPV) and hierarchical sensitivity (hSens). Hierarchical metrics were calculated per sample with values ranging from 0 to 1.0, and then averaged across all samples. The schematic shows level predictions where the “root node” is the broadest category, and not counted toward the calculation. (a) Illustrates 100% hPPV and hSens; (b) illustrates 100% hPPV and 67% hSens; (c) illustrates 67% hPPV and 67% hSens; and (d) illustrates 100% hPPV and 100% hSens, where the model made a more granular prediction than “truth” diagnosis. For example, we commonly receive specimens with a diagnosis of “metastatic carcinoma, consistent with known breast primary” without specifying the subtype. Most likely the cancer was subtyped, but the information wasn’t supplied to us. [file crc-25-0171_supplementary_figure_s3_suppsf3.pdf]
