# Supplementary material for: GPSai: A Clinically Validated AI Tool for Tissue of Origin Prediction during Routine Tumor Profiling
Source: Cancer Res Commun. 2025 Sep 1;5(9):1477–89. doi: 10.1158/2767-9764.CRC-25-0171 (PMC12399951; doi:10.1158/2767-9764.CRC-25-0171)

Supplementary Figure S4. GPSai model scoring. A representative example of scoring assigned by GPSai is shown. Subcategories (e.g., lung adenocarcinoma, lung squamous cell carcinoma) must sum to unity of the major category score (e.g., non-small cell lung carcinoma).

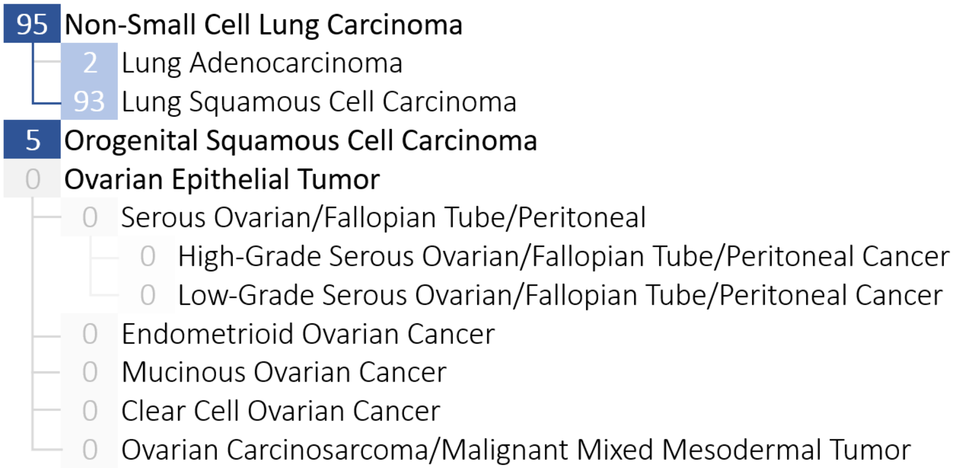

Supplement: Supplementary Figure S4 — GPSai model scoring. A representative example of scoring assigned by GPSai is shown. Subcategories (e.g., lung adenocarcinoma, lung squamous cell carcinoma) must sum to unity of the major category score (e.g., non-small cell lung carcinoma). [file crc-25-0171_supplementary_figure_s4_suppsf4.pdf]
