# Supplementary material for: GPSai: A Clinically Validated AI Tool for Tissue of Origin Prediction during Routine Tumor Profiling
Source: Cancer Res Commun. 2025 Sep 1;5(9):1477–89. doi: 10.1158/2767-9764.CRC-25-0171 (PMC12399951; doi:10.1158/2767-9764.CRC-25-0171)

Supplementary Figure S5. Threshold selection for GPSai. The dashed line at 0.55 indicates the threshold determined to optimize both CUP call rate (green line) and hierarchical positive predictive value (hPPV, blue line) for metastatic cases. A GPS score ≥0.55 indicates high confidence in the tumor type prediction and will be included on the molecular report.

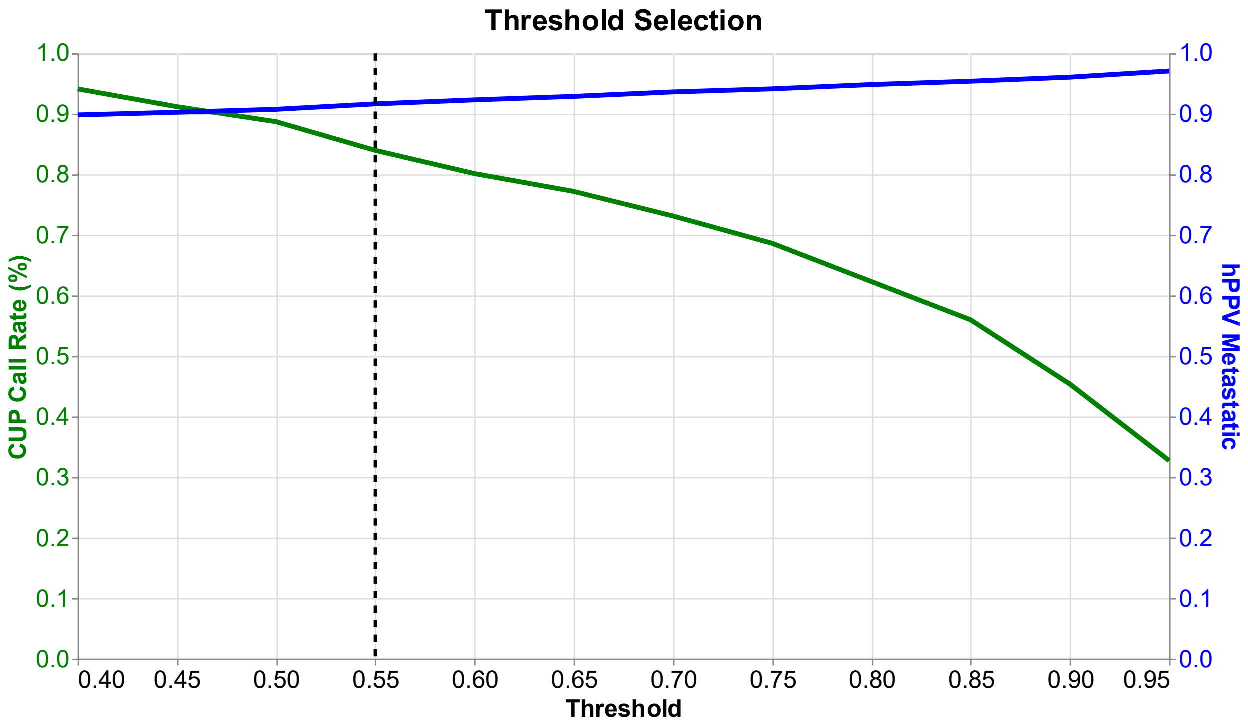

Supplement: Supplementary Figure S5 — Threshold selection for GPSai. The dashed line at 0.55 indicates the threshold determined to optimize both CUP call rate (green line) and hierarchical positive predictive value (hPPV, blue line) for metastatic cases. A GPS score ≥0.55 indicates high confidence in the tumor type prediction and will be included on the molecular report. [file crc-25-0171_supplementary_figure_s5_suppsf5.pdf]
