# Supplementary material for: GPSai: A Clinically Validated AI Tool for Tissue of Origin Prediction during Routine Tumor Profiling
Source: Cancer Res Commun. 2025 Sep 1;5(9):1477–89. doi: 10.1158/2767-9764.CRC-25-0171 (PMC12399951; doi:10.1158/2767-9764.CRC-25-0171)

Supplementary Figure S9. Changes in drug eligibility based on GPSai results. Based on diagnosis & biomarker or diagnosis-only, all cases with added eligibility **(A)** or ineligibility **(B)** for the indicated drugs.

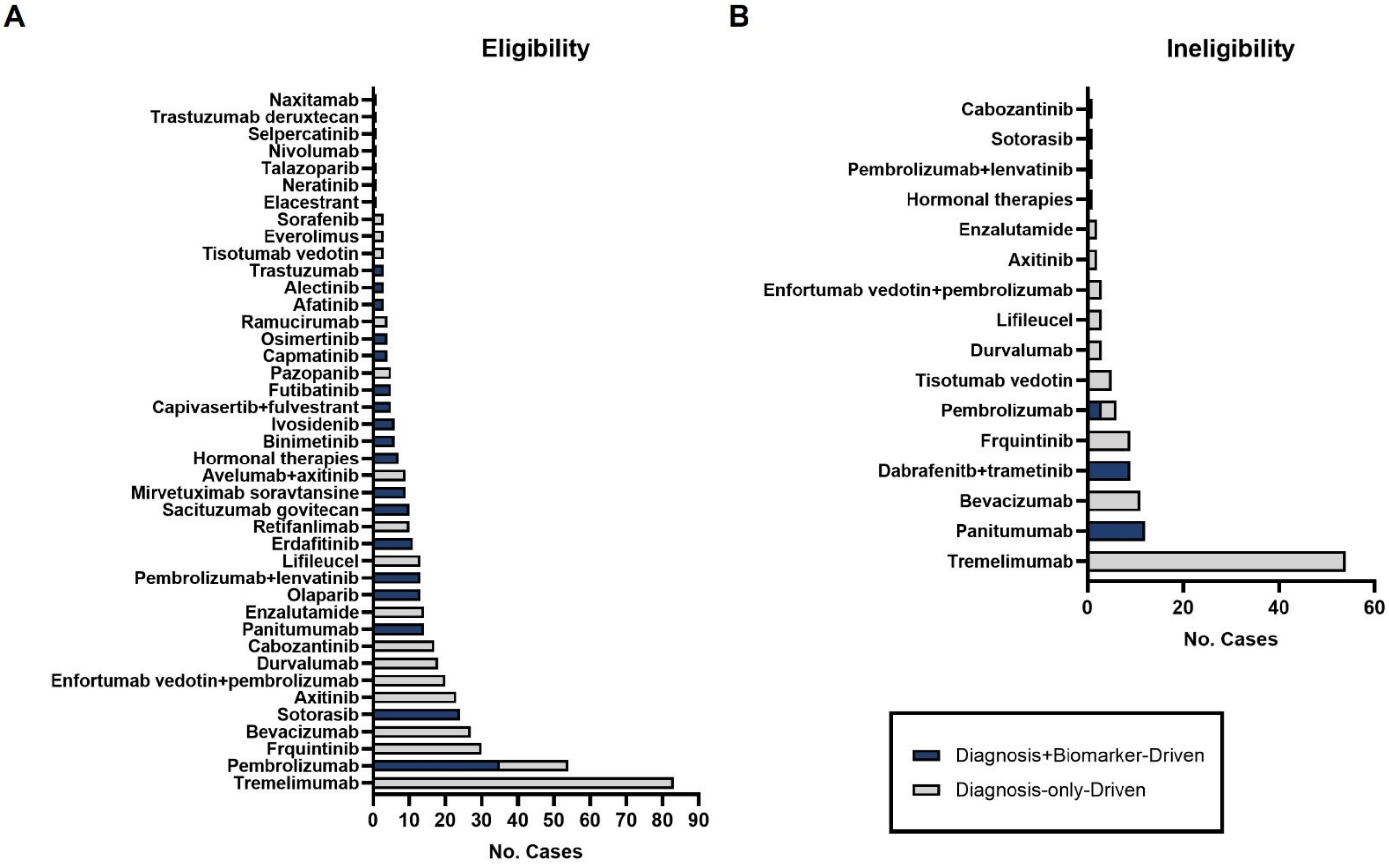

Supplement: Supplementary Figure S9 — Changes in drug eligibility based on GPSai results. Based on diagnosis & biomarker or diagnosis-only, all cases with added eligibility (a) or ineligibility (b) for the indicated drugs. [file crc-25-0171_supplementary_figure_s9_suppsf9.pdf]
