# Supplementary material for: GPSai: A Clinically Validated AI Tool for Tissue of Origin Prediction during Routine Tumor Profiling
Source: Cancer Res Commun. 2025 Sep 1;5(9):1477–89. doi: 10.1158/2767-9764.CRC-25-0171 (PMC12399951; doi:10.1158/2767-9764.CRC-25-0171)
Supplement: Supplementary Table S1 — Pathology procedures for review of GPSai results. [file crc-25-0171_supplementary_table_s1_suppst1.pdf]

Supplementary Table S1. Pathology procedures for review of GPSai results

		"Critical Value Discrepancy"	
	**Do not include on report**	**Include on report (No lineage/diagnosis change)**	**Include on report (with lineage/diagnosis change)**
**CUP**	GPSai score ≤0.55	GPSai score is ≥90% and results cannot be proven via orthogonal methods, or when there is more than one MI GPSai result with none ≥90%	GPSai score ≥90% and the result is provable by orthogonal evidence
**Non-CUP**	GPSai score ≤0.55	Submitted diagnosis has GPSai score of 0% or a single MI GPSai category has a score ≥90%, but results cannot be proven by orthogonal evidence	Submitted diagnosis has a GPSai score of 0% or a single GPSai category has a score ≥90%, and results are proven by orthogonal evidence
