# Supplementary material for: GPSai: A Clinically Validated AI Tool for Tissue of Origin Prediction during Routine Tumor Profiling
Source: Cancer Res Commun. 2025 Sep 1;5(9):1477–89. doi: 10.1158/2767-9764.CRC-25-0171 (PMC12399951; doi:10.1158/2767-9764.CRC-25-0171)
Supplement: Supplementary Table S2 — GPSai model performance in metastatic sites. [file crc-25-0171_supplementary_table_s2_suppst2.pdf]

Supplementary Table S2. GPSai model performance in metastatic sites

	Samples, N	Call Rate, %	[a]hPPV, %	[a]hSens, %	TOP1 PPV, %	TOP2 PPV, %
**Lymph node**	1578	94.0	92.1	91.6	93.3	97.6
**Liver**	2048	96.6	93.1	92.5	94.9	97.4
**Lung**	751	94.0	91.5	91.2	92.1	97.2
**Bone**	630	96.3	94.3	94.2	94.7	97.7
**Brain**	324	95.1	92.5	91.9	93.2	97.4

[a]Hierarchical metrics are reported due to the hierarchical nature of the diagnostic labels (**Supplementary Fig. S1**). A description of how the hierarchical metrics were calculated is shown in **Supplementary Fig. S3**. TOP1 = top major category selected by MI GPSai model; TOP2 = top 2 major categories predicted by the model.

Abbreviations: hPPV=hierarchical positive predictive value; hSens=hierarchical sensitivity.
