# Supplementary material for: GPSai: A Clinically Validated AI Tool for Tissue of Origin Prediction during Routine Tumor Profiling
Source: Cancer Res Commun. 2025 Sep 1;5(9):1477–89. doi: 10.1158/2767-9764.CRC-25-0171 (PMC12399951; doi:10.1158/2767-9764.CRC-25-0171)
Supplement: Supplementary Table S3 — GPSai model performance in prospective validation. [file crc-25-0171_supplementary_table_s3_suppst3.pdf]

Supplementary Table S3. GPSai model performance in prospective validation						
	[a]Samples, N	Call Rate, %	[b]hPPV, %	[b]hSens, %	TOP1 PPV, %	TOP2 PPV, %
**Non-CUP**						
**Global**	72,421	95.9	93.5	92.7	95.4	98.7
**Primary**	44,646	96.9	94.4	93.6	96.4	99.2
**Metastatic**	27,775	94.4	92.0	91.2	93.8	97.8
**CUP**	3,850	82.1	N/A	N/A	N/A	N/A

[a]The total sum of non-CUP and CUP is less than the 80,308 total cases in this prospective cohort due to a subset of cases not having submitted tumor type labels mapping to a GPSai tumor type label.

[b]Hierarchical metrics are reported due to the hierarchical nature of the diagnostic labels (**Supplementary Fig. S1**). A description of how the hierarchical metrics were calculated is shown in **Supplementary Fig. S3**. TOP1 = top one major category selected by GPSai model. TOP2 = top two major categories selected by the model. PPV is reported for TOP1 and TOP2.

Abbreviations: hPPV=hierarchical positive predictive value; hSens=hierarchical sensitivity.
