# Supplementary material for: GPSai: A Clinically Validated AI Tool for Tissue of Origin Prediction during Routine Tumor Profiling
Source: Cancer Res Commun. 2025 Sep 1;5(9):1477–89. doi: 10.1158/2767-9764.CRC-25-0171 (PMC12399951; doi:10.1158/2767-9764.CRC-25-0171)
Supplement: Supplementary Table S4 — Cases with diagnosis change due to GPSai results. [file crc-25-0171_supplementary_table_s4_suppst4.pdf]

Supplementary Table S4. Cases with diagnosis change due to GPSai results

Row Labels	Count of Submitted Lineage
**CUP**	**404**
Lung Non-small cell lung cancer (NSCLC)	105
Colorectal adenocarcinoma	38
Ovarian Surface Epithelial Carcinomas	28
Cholangiocarcinoma	25
Breast carcinoma	22
Pancreatic Adenocarcinoma	21
Kidney cancer	18
Bladder carcinoma - urothelial	18
Squamous Cell Skin Cancer	11
Mesothelioma	11
Thymic Carcinoma	9
Prostatic Adenocarcinoma	9
Neuroendocrine carcinoma	9
Melanoma	9
Gastric Adenocarcinoma	8
Endometrial carcinoma	8
Hepatocellular Carcinoma	8
Esophagogastric Junction Carcinoma	7
HNSCC	4
Soft tissue sarcoma	4
Salivary gland carcinoma	4
Cervical carcinoma	3
Thyroid carcinoma	3
Uterine Serous Carcinoma	3
Testicular carcinoma	3
Merkel Cell Carcinoma (MCC)	3
Anal Carcinoma	2
Lung Small Cell Cancer (SCLC)	2
Glioblastoma	2
SCC of rectal region	1
Lymphoma	1
Endometrial Stromal Sarcoma	1
Ewing Sarcoma	1
Penile carcinoma	1
Vulvar carcinoma	1
Male Genital Tract Malignancy	1

**Lung Non-small cell lung cancer (NSCLC)**	**87**
Bladder carcinoma - urothelial	11
HNSCC	10
Mesothelioma	9
Squamous Cell Skin Cancer	7
Lung Small Cell Cancer (SCLC)	6
Thymic Carcinoma	5
Breast carcinoma	5
Colorectal adenocarcinoma	4
Endometrial carcinoma	4
Esophagogastric Junction Carcinoma	3
Kidney cancer	3
Soft tissue sarcoma	3
Neuroendocrine carcinoma	3
Thyroid carcinoma	3
Melanoma	3
Hepatocellular Carcinoma	2
Prostatic Adenocarcinoma	1
Salivary gland carcinoma	1
Pancreatic Adenocarcinoma	1
Anaplastic thyroid carcinoma	1
Ovarian Surface Epithelial Carcinomas	1
Lung Non-small cell lung cancer (NSCLC) (squamous to adenocarcinoma)	1
**Colorectal adenocarcinoma**	**22**
Endometrial carcinoma	6
Neuroendocrine carcinoma	5
Prostatic Adenocarcinoma	2
Lung Non-small cell lung cancer (NSCLC)	2
Ovarian Surface Epithelial Carcinomas	1
Cholangiocarcinoma	1
Salivary gland carcinoma	1
Squamous Cell Skin Cancer	1
Anal Carcinoma	1
Pancreatic Adenocarcinoma	1
Hepatocellular Carcinoma	1
**Breast carcinoma**	**21**
Lung Non-small cell lung cancer (NSCLC)	9
Ovarian Surface Epithelial Carcinomas	3
Squamous Cell Skin Cancer	3

Bladder carcinoma - urothelial	2
Hepatocellular Carcinoma	1
Anal Carcinoma	1
Salivary gland carcinoma	1
Melanoma	1
**Neuroendocrine carcinoma**	**19**
Merkel Cell Carcinoma (MCC)	6
Breast carcinoma	5
Thyroid carcinoma	1
Lung Non-small cell lung cancer (NSCLC)	1
Melanoma	1
Neuroblastoma	1
Prostatic Adenocarcinoma	1
Hepatocellular Carcinoma	1
Uterine Sarcoma	1
Meningioma	1
**Endometrial carcinoma**	**18**
Ovarian Surface Epithelial Carcinomas	9
Lung Non-small cell lung cancer (NSCLC)	2
Cholangiocarcinoma	1
Uterine Sarcoma	1
Colorectal adenocarcinoma	1
Neuroendocrine carcinoma	1
Cervical carcinoma	1
Multiple Myeloma	1
Lymphoma	1
**Bladder carcinoma - urothelial**	**16**
Prostatic Adenocarcinoma	5
Neuroendocrine carcinoma	2
Lung Non-small cell lung cancer (NSCLC)	2
Lymphoma	1
Cervical carcinoma	1
Colorectal adenocarcinoma	1
Soft tissue sarcoma	1
Breast carcinoma	1
HNSCC	1
Endometrial carcinoma	1
**Pancreatic Adenocarcinoma**	**16**
Colorectal adenocarcinoma	5
Neuroendocrine carcinoma	3

Hepatocellular Carcinoma	1
Bladder carcinoma - urothelial	1
Osteosarcoma	1
Cholangiocarcinoma	1
Breast carcinoma	1
Melanoma	1
Prostatic Adenocarcinoma	1
Lung Non-small cell lung cancer (NSCLC)	1
**Ovarian Surface Epithelial Carcinomas**	**10**
Mesothelioma	5
Kidney cancer	1
Non Epithelial Ovarian Cancer (non-EOC)	1
Breast carcinoma	1
Uterine Serous Carcinoma	1
Melanoma	1
**Salivary gland carcinoma**	**9**
Squamous Cell Skin Cancer	7
Lung Non-small cell lung cancer (NSCLC)	1
Neuroendocrine carcinoma	1
**Cervical carcinoma**	**9**
Bladder carcinoma - urothelial	3
Ovarian Surface Epithelial Carcinomas	2
Neuroendocrine carcinoma	2
Vulvar carcinoma	1
Endometrial carcinoma	1
**Gastric Adenocarcinoma**	**8**
Cholangiocarcinoma	2
Colorectal adenocarcinoma	2
Melanoma	1
Ovarian Surface Epithelial Carcinomas	1
Pancreatic Adenocarcinoma	1
Neuroendocrine carcinoma	1
**Cholangiocarcinoma**	**7**
Lung Non-small cell lung cancer (NSCLC)	3
Colorectal adenocarcinoma	1
Hepatocellular Carcinoma	1
Neuroendocrine carcinoma	1
Pancreatic Adenocarcinoma	1
**Ovarian Surface Epithelial Carcinoma**	**6**
Esophagogastric Junction Carcinoma	1

Colorectal adenocarcinoma	1
Endometrial carcinoma	1
Melanoma	1
Thyroid carcinoma	1
Lung Non-small cell lung cancer (NSCLC)	1
**Small Bowel Adenocarcinoma**	**5**
Pancreatic Adenocarcinoma	3
Gastric Adenocarcinoma	1
Lung Non-small cell lung cancer (NSCLC)	1
**HNSCC**	**5**
Lung Non-small cell lung cancer (NSCLC)	2
Salivary gland carcinoma	1
Bladder carcinoma - urothelial	1
Ewing Sarcoma	1
**Kidney cancer**	**5**
Lung Non-small cell lung cancer (NSCLC)	3
Breast carcinoma	1
Lymphoma	1
**Hepatocellular Carcinoma**	**3**
Salivary gland carcinoma	1
Breast carcinoma	1
Lung Non-small cell lung cancer (NSCLC)	1
**Squamous Cell Skin Cancer**	**3**
Male Genital Tract Malignancy	1
HNSCC	1
Lung Non-small cell lung cancer (NSCLC)	1
**Melanoma**	**3**
Soft tissue sarcoma	2
Osteosarcoma	1
**Thyroid Carcinoma**	**3**
Lung Non-small cell lung cancer (NSCLC)	2
Kidney cancer	1
**Spindle cell sarcoma**	**2**
Liposarcoma	2
**Lung Small Cell Cancer (SCLC)**	**2**
Lung Non-small cell lung cancer (NSCLC)	1
Merkel Cell Carcinoma (MCC)	1
**Prostatic Adenocarcinoma**	**2**
Cholangiocarcinoma	1
Osteosarcoma	1

**Esophagogastric Junction Carcinoma**	**2**
Lung Non-small cell lung cancer (NSCLC)	1
Ovarian Surface Epithelial Carcinomas	1
**Soft tissue sarcoma**	**2**
Meningioma	1
Squamous Cell Skin Cancer	1
**Esophageal carcinoma**	**2**
Lung Non-small cell lung cancer (NSCLC)	1
Neuroendocrine carcinoma	1
**Vulvar Squamous Cell Carcinoma**	**2**
Uterine Serous Carcinoma	1
Vulvar carcinoma	1
**Prostate Adenocarcinoma**	**2**
Colorectal adenocarcinoma	1
Meningioma	1
**Ovarian Surface Epithelial Carcinoma non-clear cell**	**1**
Ovarian Surface Epithelial Carcinomas	1
**Uterine Serous Carcinoma**	**1**
Ovarian Surface Epithelial Carcinomas	1
**Bladder carcinoma - non-urothelial**	**1**
Bladder carcinoma - urothelial	1
**Sarcoma**	**1**
Melanoma	1
**Ependymoma**	**1**
Astroblastoma	1
**Germ cell tumor**	**1**
Hepatocellular Carcinoma	1
**Glioblastoma**	**1**
Lung Non-small cell lung cancer (NSCLC)	1
**Astrocytoma**	**1**
Glioblastoma	1
**Low Grade Glioma**	**1**
Malignant Solitary Fibrous Tumor of the Pleura (MSFT)	1
**Grand Total**	**704**
