# Supplementary material for: GPSai: A Clinically Validated AI Tool for Tissue of Origin Prediction during Routine Tumor Profiling
Source: Cancer Res Commun. 2025 Sep 1;5(9):1477–89. doi: 10.1158/2767-9764.CRC-25-0171 (PMC12399951; doi:10.1158/2767-9764.CRC-25-0171)
Supplement: Supplementary Table S5 — Fusions as orthogonal evidence in cases with ‘Critical Value Discrepancies’. [file crc-25-0171_supplementary_table_s5_suppst5.pdf]

Supplementary Table S5. Fusions as orthogonal evidence in cases with 'Critical Value Discrepancies'

Fusion	Supports Submitted Diagnosis	Supports GPSai Prediction
CD74:ROS1	0	1
ESR1:TNRC6B	0	1
FGFR2:WEE1	0	1
KIF5B:RET	0	1
PGR:NR4A3	0	1
PTPRK:RSPO3	0	2
TMPRSS2:ERG	2	3
EML4:ALK	1	0
EWSR1:FLI1	1	0
**Total**	**4**	**10**
