# Supplementary material for: GPSai: A Clinically Validated AI Tool for Tissue of Origin Prediction during Routine Tumor Profiling
Source: Cancer Res Commun. 2025 Sep 1;5(9):1477–89. doi: 10.1158/2767-9764.CRC-25-0171 (PMC12399951; doi:10.1158/2767-9764.CRC-25-0171)
Supplement: Supplementary Table S6 — Physician survey results. [file crc-25-0171_supplementary_table_s6_suppst6.pdf]

| Supplementary Table S6. Physician survey results | | | | |
|---|---|---|---|---|
| | **Yes** | **No** | **Other** | **Unsure/Pt. Did Not Return to Clinic** |
| **Did the treating oncologist or pathologist accept the GPSai tissue of origin/lineage change?** | 87 | 10 | N/A | N/A |
| **Did the GPSai results assist in making a diagnosis?** | 86 | 10 | 1 | N/A |
| **Did the GPSai results change the treatment plan for this patient?** | 52 | 36 | N/A | 9 |
| **Is there reasonable expectation of clinical benefit due to this change?** | 42 | 1 | N/A | 18 |
| **Did the test results help the patient become eligible for a clinical trial?** | 8 | 82 | N/A | N/A |
