# Supplementary material for: GPSai: A Clinically Validated AI Tool for Tissue of Origin Prediction during Routine Tumor Profiling
Source: Cancer Res Commun. 2025 Sep 1;5(9):1477–89. doi: 10.1158/2767-9764.CRC-25-0171 (PMC12399951; doi:10.1158/2767-9764.CRC-25-0171)

Supplementary Figure S6. Performance per category for all categories (primary tumors). Confusion matrix for primary tumors in retrospective validation cohort. Positive predictive value (PPV) for each category is indicated by the bars above and sensitivity by the bars to the right. Numbers of each predicted tumor type are shown in the boxes, with darker shading of the diagonal indicating a higher number of correctly predicted tumors (matching outside label).

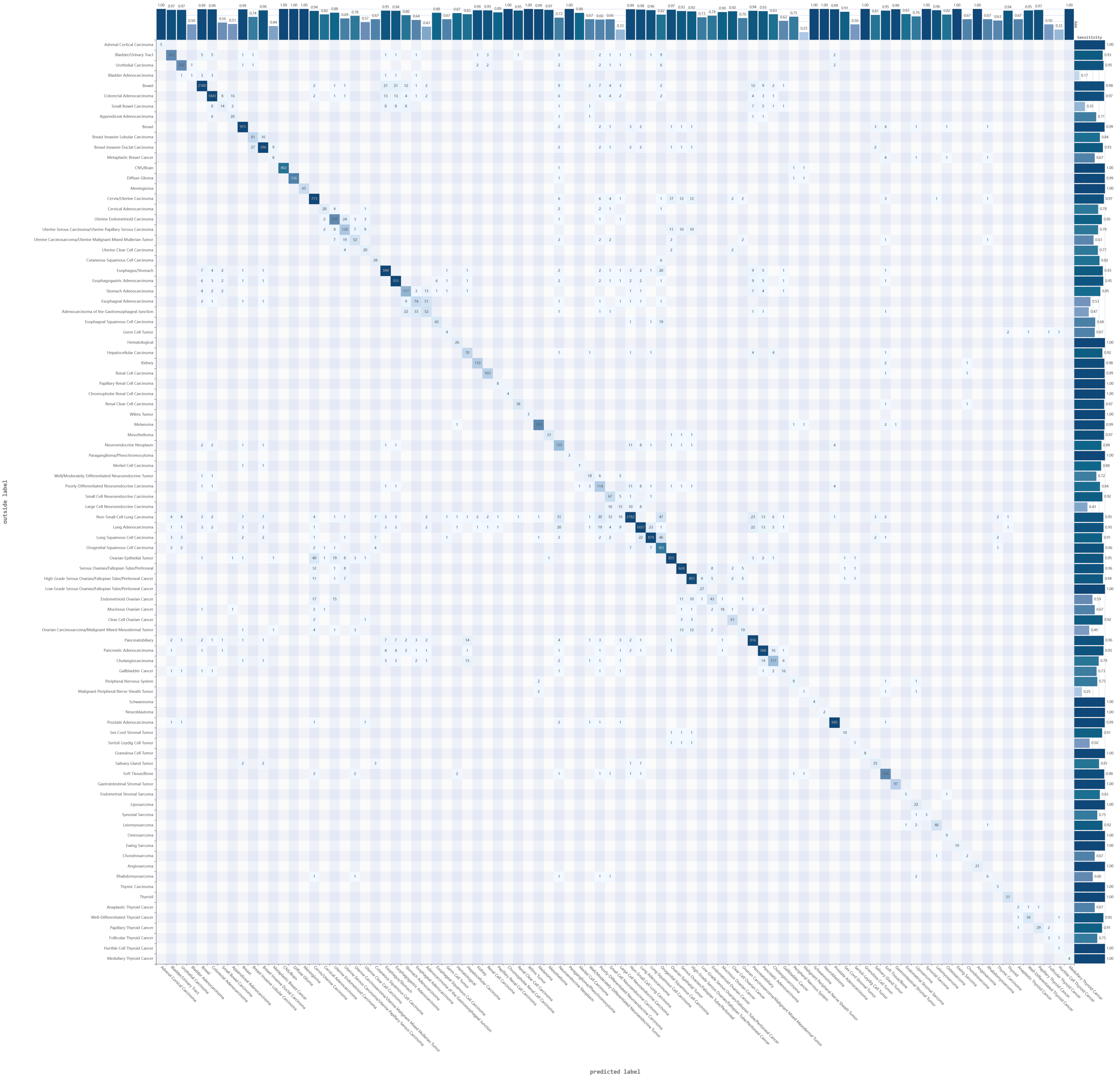

Supplement: Supplementary Figure S6 — Performance per category for all categories (primary tumors). Confusion matrix for primary tumors in retrospective validation cohort. Positive predictive value (PPV) for each category is indicated by the bars above and sensitivity by the bars to the right. Numbers of each predicted tumor type are shown in the boxes, with darker shading of the diagonal indicating a higher number of correctly predicted tumors (matching outside label). [file crc-25-0171_supplementary_figure_s6_suppsf6.pdf]
