# Supplementary material for: GPSai: A Clinically Validated AI Tool for Tissue of Origin Prediction during Routine Tumor Profiling
Source: Cancer Res Commun. 2025 Sep 1;5(9):1477–89. doi: 10.1158/2767-9764.CRC-25-0171 (PMC12399951; doi:10.1158/2767-9764.CRC-25-0171)

Supplementary Figure S7. Performance per category for all categories (metastatic tumors). Confusion matrix for metastatic tumors in retrospective validation cohort. Positive predictive value (PPV) for each category is indicated by the bars above and sensitivity by the bars to the right. Numbers of each predicted tumor type are shown in the boxes, with darker shading of the diagonal indicating a higher number of correctly predicted tumors (matching outside label).

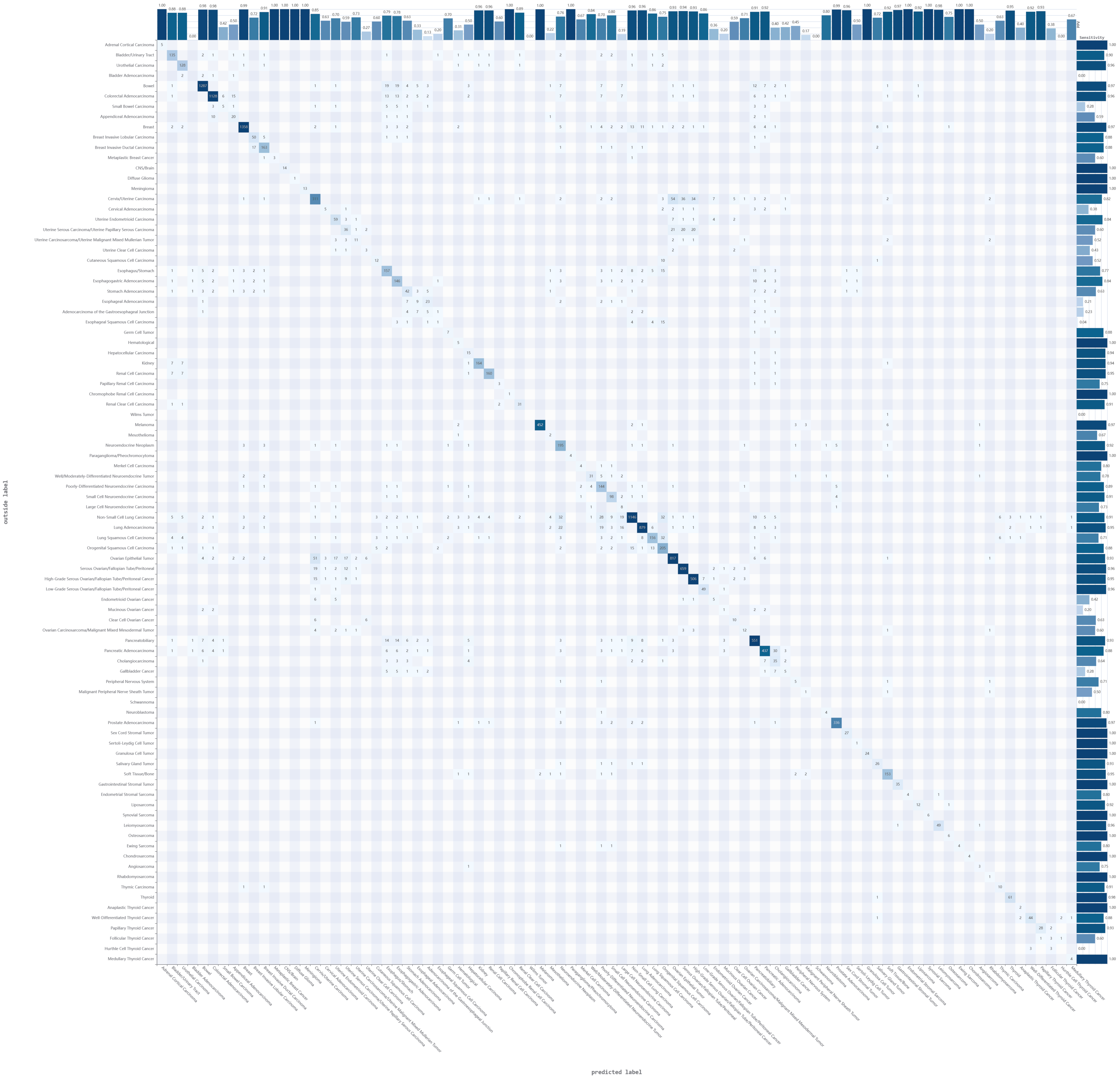

Supplement: Supplementary Figure S7 — Performance per category for all categories (metastatic tumors). Confusion matrix for metastatic tumors in retrospective validation cohort. Positive predictive value (PPV) for each category is indicated by the bars above and sensitivity by the bars to the right. Numbers of each predicted tumor type are shown in the boxes, with darker shading of the diagonal indicating a higher number of correctly predicted tumors (matching outside label). [file crc-25-0171_supplementary_figure_s7_suppsf7.pdf]
