# Supplementary material for: GPSai: A Clinically Validated AI Tool for Tissue of Origin Prediction during Routine Tumor Profiling
Source: Cancer Res Commun. 2025 Sep 1;5(9):1477–89. doi: 10.1158/2767-9764.CRC-25-0171 (PMC12399951; doi:10.1158/2767-9764.CRC-25-0171)

Supplementary Figure S8. Prospective validation. Model performance per major cancer category is shown for primary and metastatic tumors (N = 72,421). Confusion matrix shows numbers of each predicted tumor type in the boxes, with numbers of correctly predicted tumors (matching submitted diagnosis) in each category shown in the diagonal. Positive predictive value (PPV) for each category is indicated by the bars above and sensitivity by the bars to the right.

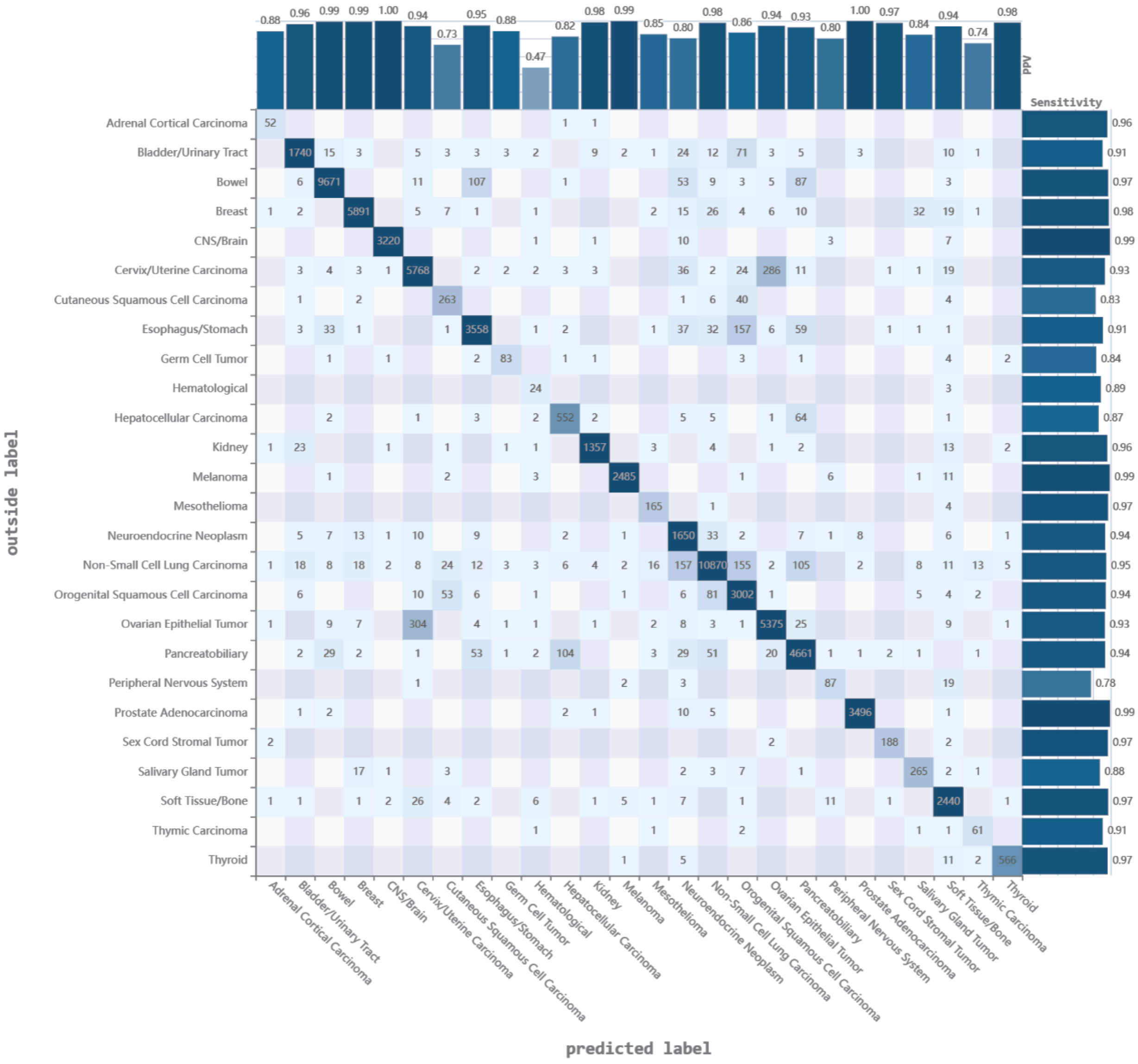

Supplement: Supplementary Figure S8 — Prospective validation. Model performance per major cancer category is shown for primary and metastatic tumors (N = 72,421). Confusion matrix shows numbers of each predicted tumor type in the boxes, with numbers of correctly predicted tumors (matching submitted diagnosis) in each category shown in the diagonal. Positive predictive value (PPV) for each category is indicated by the bars above and sensitivity by the bars to the right. [file crc-25-0171_supplementary_figure_s8_suppsf8.pdf]
